# Achieving Ah-Level Zn–MnO_2_ Pouch Cells via Interfacial Solvation Structure Engineering

**DOI:** 10.1007/s40820-025-01935-6

**Published:** 2026-01-02

**Authors:** Jing Wei, Lichao Tan, Qianyi Ma, Xintao Long, Shibin Li, Yu Shi, Rui Gao, Zijing Xu, Dan Luo, Jie Zhang, Dagang Li, Xin Wang, Aiping Yu, Zhongwei Chen

**Affiliations:** 1 Institute of Carbon Neutrality, Zhejiang Wanli University, Ningbo, 315100 People’s Republic of China; 2 Department of Chemical Engineering, Waterloo Institute for Nanotechnology, University of Waterloo, Waterloo, N2L 3G1 Canada; 3 Power Battery and Systems Research Center, State Key Laboratory of Catalysis, Dalian Institute of Chemical Physics, Chinese Academy of Sciences, Dalian, 116023 People’s Republic of China; 4 College of Material Science and Engineering, Nanjing Forestry University, Nanjing, 210037 People’s Republic of China

**Keywords:** Aqueous zinc-ion batteries, In situ spectroscopy, Interfacial solvation structure, Nanocellulose

## Abstract

Aqueous zinc-ion batteries (AZIBs) offer a safe, cost-effective, and high-capacity energy storage solution, yet their performance is hindered by interfacial challenges at the Zn anode, including hydrogen evolution, corrosion, and dendritic Zn growth. While most studies focus on regulating Zn^2+^ solvation structures in bulk electrolytes, the evolution of interfacial solvation—where Zn^2+^ undergoes desolvation and deposition—remains insufficiently explored. Here, we introduce sulfated nanocellulose (SNC), an anion-rich biopolymer, to tailor the interfacial solvation structure without altering the bulk electrolyte composition. Using in situ attenuated total reflection Fourier transform infrared spectroscopy and fluorescence interface-extended X-ray absorption fine structure, we reveal that SNC facilitates the formation of a low-coordinated Zn^2+^ solvation shell at the interface by weakening H_2_O coordination. This transformation is driven by electrostatic interactions between Zn^2+^ and anchored sulfate groups, thereby reducing water activity, improving interfacial stability during charge/discharge, and suppressing parasitic reactions. Consequently, a high average coulombic efficiency of 99.6% over 500 cycles in Zn|Ti asymmetric cells and 1.5 Ah pouch cells (13.4 mg cm^−2^ loading, remained stable over 250 cycles) were achieved in SNC-induced AZIBs. This work underscores the importance of interfacial solvation structure engineering—beyond traditional bulk electrolyte design—in enabling practical, high-performance AZIBs.

**Supplementary Information:**

The online version contains supplementary material available at 10.1007/s40820-025-01935-6.

## Highlights

- This work introduces sulfated nanocellulose as an anion-rich additive to tailor the Zn anode interfacial solvation structure, reducing interfacial H_2_O activity and suppressing hydrogen evolution.
- In-situ attenuated total reflection Fourier transform infrared and fluorescence interface-extended X-ray absorption fine structure reveal the formation of a low-coordination Zn^2+^ solvation shell at the interface, facilitating rapid desolvation kinetics, enhancing interfacial stability during cycling.
- Practical aqueous Zn–MnO_2_ pouch cells (1.5 Ah), underscoring the potential of interfacial solvation engineering for high-performance aqueous zinc-ion batteries.

**Supplementary Information:**

The online version contains supplementary material available at 10.1007/s40820-025-01935-6.

## Graphical abstract

**Figure:**
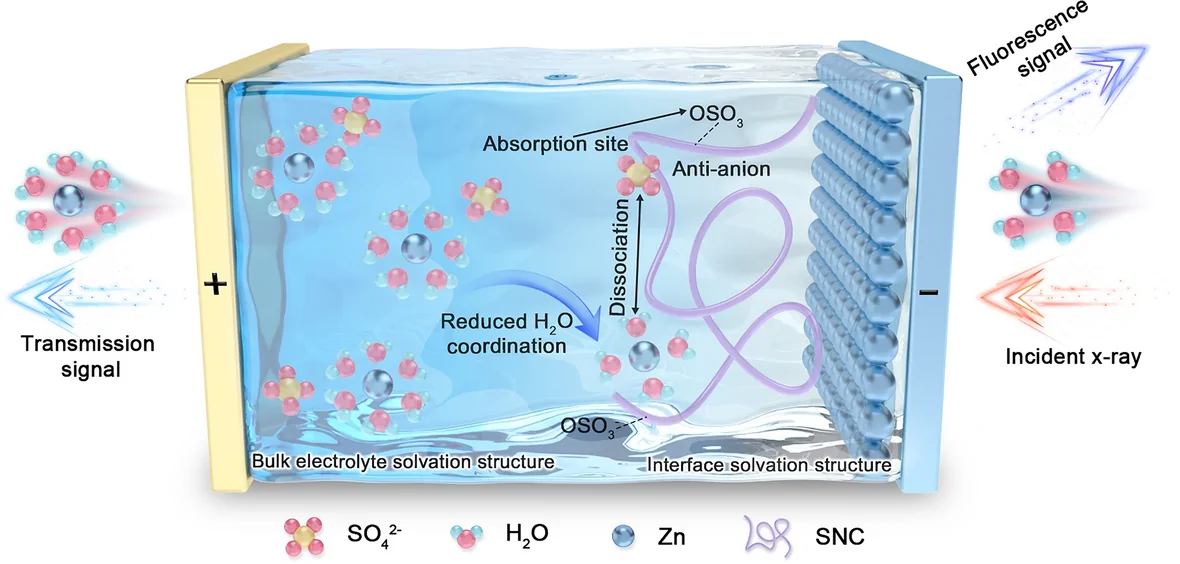

## Introduction

The ever-growing demand for energy storage devices and portable power supplies has spurred the quest for post-lithium-ion batteries [1, 2]. Due to their intrinsic safety, cost-effectiveness (≈2 USD kg^−1^, metal zinc), low redox potential (− 0.76 V vs. standard hydrogen electrode), and high theoretical capacity (820 mAh g^−1^ and 5854 mAh cm^−2^), zinc (Zn) anodes have attracted considerable attention in state-of-the-art energy storage systems [3–7]. However, aqueous electrolytes have a narrow thermodynamic stability window of 1.23 V, and metals with lower redox potentials theoretically are unsuitable for use as anodes in aqueous-based electrolytes. Like lithium (Li) metal batteries, aqueous zinc-ion batteries (AZIBs) face significant challenges due to parasitic side reactions [8, 9]. During the repeated Zn plating/stripping processes, hydrogen evolution reaction (HER), Zn metal corrosion, and morphological variations shorten the cycle life, leading to rapid battery failure [10]. Therefore, efforts to explore strategies including solid electrolyte interphase construction, separator modification, composite anode re-preparation, and electrolyte solvation design to develop ultra-stable AZIBs have been revived [6, 11–14].

In the process of practical application, HER is a serious obstacle that seriously affects the practical application of aqueous pouch cells. Due to its lack of a rigid shell-like prismatic cells, HER-induced gas evolution causes pouch cells to expand, ultimately leading to structural rupture and failure. Therefore, HER suppression is an important factor in promoting the practical application of AZIBs [15, 16]. In this regard, (aminomethyl)phosphonic acid (AMPA) additives act as a barrier to water dissociation (i.e., spontaneous corrosion) by preferentially adsorbing on the surface of the zinc anode to inhibit the growth of zinc dendrites and achieve highly reversible and stable zinc anodes [17]. However, the solvated water molecules around the zinc ion are also considered to be the cause of HER during zinc deposition. At present, the solvation structure design of AZIBs including bridging solvation structure, eutectic solvation shell, and weakly solvating electrolyte has greatly improved their cycle life and the reversibility of the Zn anode [18–22]. It is worth noting that compared with the bulk electrolyte structure design, the solvation structure at the Zn anode interface is a critical factor in facilitating Zn plating because deposited Zn originates directly from the interfacial solvation structure. Constantly, it is essential to build the low-coordinated Zn-ion solvation structure to regulate interfacial H_2_O activity and reduce the coordination number in the Zn-ion solvation structure to suppress the HER [23, 24].

Herein, we tailored a low-coordination interface Zn-ion solvation structure with an anion-rich sulfated nanocellulose (SNC) additive. The interfacial solvation structures were further studied through in situ attenuated total reflection Fourier transform infrared (ATR-FTIR) and fluorescence interface-extended X-ray absorption fine structure (FI-EXAFS). The SNC molecules with OSO_3_^−^ form a liquid/solid hybrid layer at the Zn metal anode interface, controlling the content of H_2_O molecules, which constructs the low-coordinated interface Zn-ion solvation structure. As a result, an average CE of 99.6% over 500 cycles was achieved at 5 mA cm^−2^ in Zn|Ti asymmetric cells, and long-term cycling stability over 1300 h confirmed the high reversibility in Zn|Zn symmetric cells. The 1.5 Ah pouch cell demonstrates the practical potential of this strategy for 250 cycles.

## Experimental Section

### Electrolyte Preparation

The aqueous electrolytes were 2 M ZnSO_4_ in deionized water with/without SNC (0, 0.5, 1, 5, 10 mg mL^−1^), which were donated as ZnSO_4_ (also donated as BE), ZnSO_4_-SNC0.5, ZnSO_4_-SNC1 (also donated as BE + SNC), and ZnSO_4_-SNC5, ZnSO_4_-SNC10, respectively.

### Material Characterization

The surface morphologies of the Zn anode and MnO_2_/CNT cathode were characterized by Hitachi SU5000 field emission scanning electron microscope (FESEM). The in situ optical images of zinc anode were obtained on a Bruker Innova Atomic Force Microscope (AFM) by using a home-made electrochemical cell. The applied current density is 10 mA cm^−2^. The synchrotron two-dimensional synchrotron X-ray diffraction (GIXRD) images and patterns of the Zn anode were performed on VESPERS beamline at the Canadian Light Sources. The energy of X-ray beam used for GIXRD is 11 keV. X-ray laminography were collected on BMIT-BM beamline with the filtered white beam. A white beam microscope (Optique Peter) coupling with a sCMOS camera Edge 5.5 (PCO) set at 5 × magnification was used for the scans with the pixel size of 1.44 µm. Before measurement, sample stage was calibrated by using regular X-ray micro-CT and then change the rotation axis to 60 degrees for Laminograph test. The chemistry bonds were investigated by Bruker Optics Vertex 70 Fourier transform infrared (FTIR) spectrometer. The H_2_ released during Zn deposition was quantified via in situ electrochemical gas chromatography (EC-GC) and CHI660E electrochemical workstation. X-ray photoelectron spectroscopy (XPS) was tested by Thermo Scientific ESCALAB Xi^+^, which was acquired by utilizing an Al K α (*λ* = 0.83 nm, hν = 1486.7 eV) X–ray source operated at 2 kV and 20 mA to record C 1 s on the surface of the Zn anode in the Zn|Zn symmetric cell after cycled with SNC additive. The element composition of the cycled Zn was explored by using time-of-flight secondary ion mass spectrometry (TOF–SIMS, ION TOF) with a Cs^+^ ion beam. The in situ attenuated total reflection surface-enhanced Infrared absorption spectroscopy also known as ATR-FTIR spectroscopy was used to probe additional parasitic mechanisms during different potentials. Each spectrum was recorded at a spectral resolution of 4 cm^−1^ with a time resolution of 15 s. XRD was employed to analyze the crystal structure of the MnO_2_/CNT samples collected on an X’Pert Pro X-ray diffractometer with Cu Kα radiation (*λ* = 1.5418 Å).

More details of other syntheses and characterizations can be seen in Supporting Information.

## Results and Discussion

### Interface Solvation Structure Construction

The hydrogen (H_2_) evolution is the obstacle to building AZIBs with high Zn^2+^/Zn reversibility and long cycling stability in 2 M ZnSO_4_ aqueous baseline electrolyte (named as BE) due to its higher redox potential when compared with Zn deposition [25]. Coulombic efficiency (CE) is an important performance parameter that reflects the potential practical application value of electrolytes. Through basic experiments, we find that 1 wt% SNC in 2 M ZnSO_4_ (denoted as ZnSO_4_-SNC1 in Figs. S1 and S2) has the highest reversibility of Zn plating/stripping process, minimum corrosion current, and excellent cycling stability when compared with BE and other electrolytes with different SNC contents. Owing to "volume effect" in the liquid/solid hybrid layer, the SNC molecules are not only involved in coordinating with Zn^2+^ in the electrolyte, but also accumulate at the anode surface to form a semi-ordered interphase. It can significantly reduce the content of H_2_O at the interface, where H_2_O is replaced by SNC. Reducing the H_2_O content at the Zn metal interface can effectively suppress the HER, enhancing the stability of the Zn anode. Since SNC itself has abundant OH, it can weaken H_2_O–H_2_O hydrogen bonds, which destabilize metallic Zn in bulk electrolytes and disrupt the continuous aqueous electrolyte network. These strategies minimize the activity of H_2_O and improve the overall stability of the Zn anode.

**Fig. 1:**
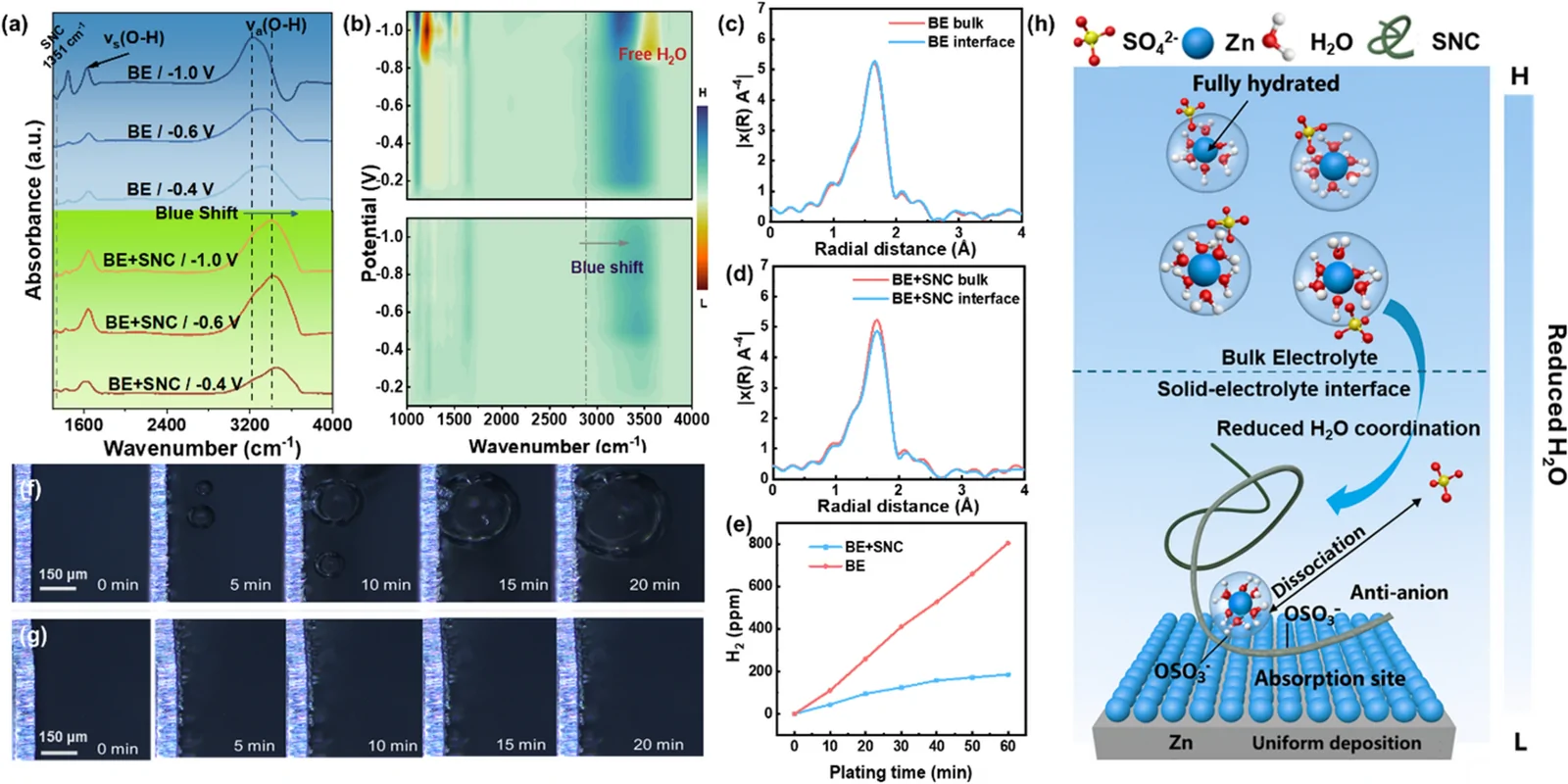
**a** In situ ATR-FTIR for Zn anode interface with the different potential (− 0.4 V, − 0.6 V, − 1.0 V); **b** intensity contrast spectrum for in situ ATR-FTIR for Zn anode interface with the different potential (− 0.4 V, − 0.6 V, − 1.0 V) in BE (top) and BE + SNC (bottom); EXAFS R-space of bulk electrolyte and interface in **c** BE; **d** BE + SNC (EXAFS R-space for the Zn-ion solvation structure in the Zn anode interface tested at 1 mA cm^−2^); **e** EC-GC for the evaluation of HER; in situ optical microscope for observing HER behaviors in **f** BE and **g** BE + SNC; **h** schematic diagram of uniform Zn deposition in the SNC interface layer to inhibit HER by promoting zinc-ion dissociation and controlling the H_2_O content of the interface layer

**Fig. 2:**
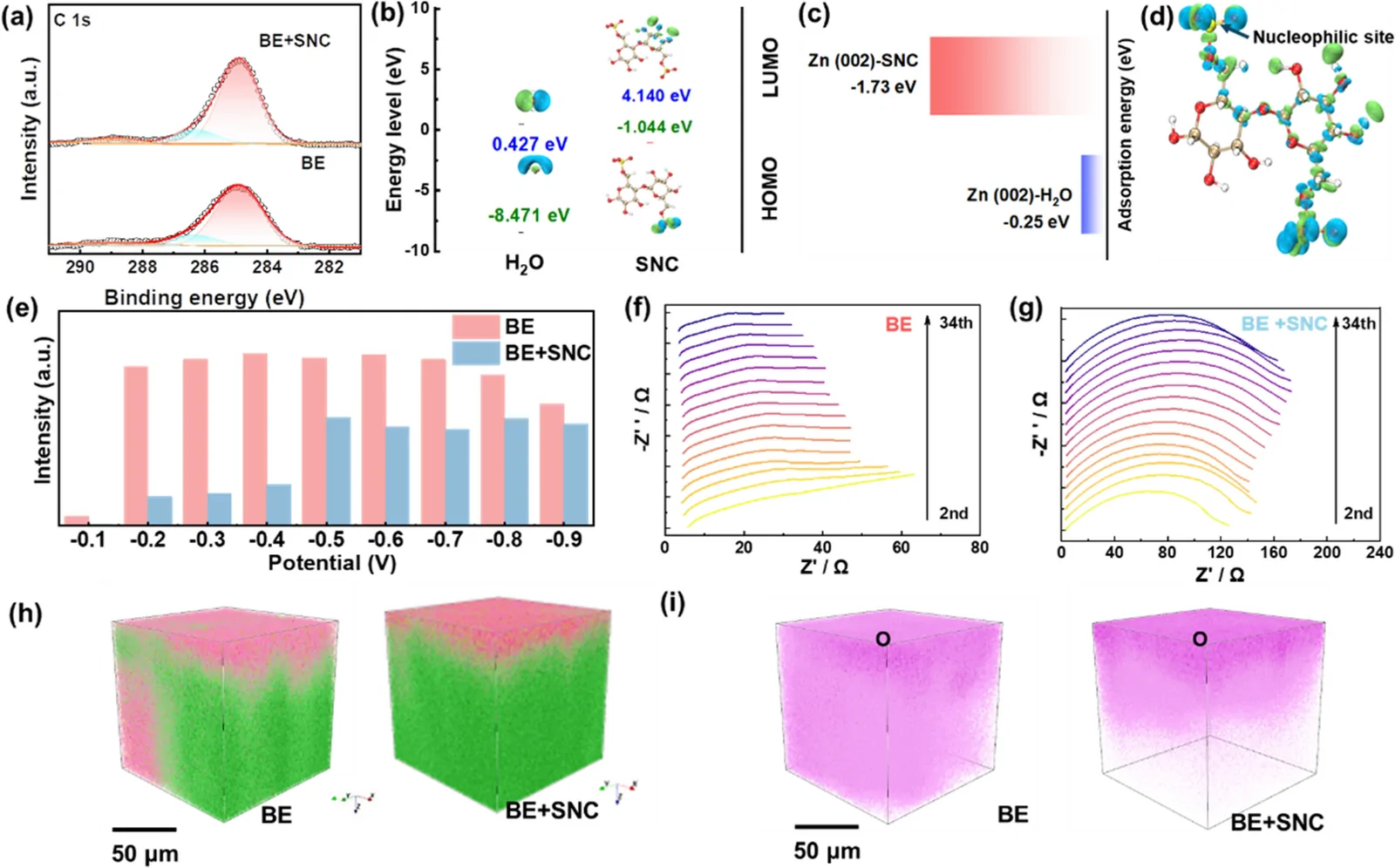
**a** XPS for C 1 s in the Zn anode surface cycled in BE and BE + SNC; **b** LUMO and HOMO with SNC and H_2_O; **c** adsorption energy of H_2_O and SNC on Zn (002) surface; **d** Schematic diagram of the nucleophilic site for SNC; **e** comparison of free H_2_O content at the Zn anode interface (obtained by FTIR integration); in situ EIS plots tested in **f** BE; and **g** BE + SNC; ToF–SIMS of Zn anode surface (red O and green Zn) **h** in total view; **i** in O element view. All the anodes were prepared after the 30th stripping/plating process at 1 mA cm^−2^ and 1 mAh cm^−2^

In situ attenuated total reflection Fourier transform infrared (ATR-FTIR) and fluorescence interface-extended X-ray absorption fine structure (FI-EXAFS) are applied to study the Zn plating and stripping interface behaviors and Zn-ion solvation structure in the different electrolytes. Electrochemical gas chromatography (EC-GC) was used to evaluate H_2_ evolution in different electrolytes. The shifts in the O–H stretching vibration v(O–H) were observed to determine the variation in H_2_O activity (Fig. 1a). Comparison of the FTIR spectra of BE and BE + SNC at − 0.4, − 0.6, and − 1.0 V, respectively, suggests that SNC has a specific peak at 1351 cm^−1^ different from BE in the wavenumber range of 1100–2000 cm^−1^. It is been proved that SNC can be adsorbed on the interface under potential, which indicates the formation of an SNC-based liquid/solid hybrid layer. The v_a_(O–H) located at 3245 cm^−1^ and v_s_(O–H) located at 1631 cm^−1^ were observed to blueshift from BE to BE + SNC at the same potential − 1.0 V, suggesting a strengthened O–H bond due to the interaction between SNC and interface H_2_O, which proves that the hydrogen bond network between H_2_O is broken in the Zn anode surface. In nuclear magnetic resonance (^1^H-NMR) spectra, the ^1^H peak of H_2_O from ZnSO_4_ shifts to lower ppm after the introduction of SNC, verifying the strong O–H bond effect between SNC and H_2_O (Fig. S3). The reduced water activity of BE + SNC was determined by integrating v_s_(O–H) peak, indicating the enrichment of interfacial H_2_O was decreased at different voltages (Fig. 2e). The SNC reduced the overall interfacial H_2_O content and destroyed the continuous hydrogen bond network of water molecules, which is beneficial to inhibiting H_2_ evolution. In the Zn plating process, a reduction of free water (manifested by the decrease in intensity) and aggregated anions (shown as intensity attenuation of SO_4_^2−^ vibration (*v*(SO_4_^2−^)) at 1080 cm^−2^) were found in the BE when increasing the potential, whereas BE + SNC does not have this phenomenon (Fig. 1b). This accelerates H_2_ evolution and electrostatic coupling intensity between SO_4_^2−^ and Zn^2+^, hindering the smooth and rapid ion diffusion and ultimately leading to poor coulombic efficiency in the aqueous BE.

To confirm the coordination of Zn^2+^ in the bulk electrolyte, density functional theory (DFT) is further applied. The binding energy between Zn^2+^ and the SNC molecule is observed to be lower than that of H_2_O (Fig. S3a). Owing to the lower adsorption energy, it is impossible for SNC to enter the inner solvation shell but remain outside the first solvation structure of the Zn ion, which is confirmed in molecular dynamics (MD) simulation (Figs. S5c and S6). The Zn ion is "closed" in the form of a "solvent cage" which is formed by hydrogen bonding between H_2_O molecules, while SO_4_^2−^ is attached around the first solvation shell in the bulk electrolyte both in BE and BE + SNC electrolyte. In the Fourier transformation of the EXAFS, a similar intensity peak attributed to Zn–O is observed at 1.60 Å both in BE and BE + SNC. This phenomenon indicates that in the bulk electrolyte with/without the addition of SNC, the Zn-ion solvation structure still keep the same (Fig. 1c, d). Furthermore, we applied FI-EXAFS to obtain the Zn K-edge EXAFS from the anode interface. Since X-ray fluorescence itself has weak penetration ability, we can attribute the collected signal to the signal at the zinc interface. With the condition of 1 mA cm^−2^, the average coordination number of Zn ions decreases significantly in the interface layer with SNC (Fig. 1d). This decrease in coordination number is consistent with the conclusion of in situ ATR-FTIR, which indicates that the number of H_2_O at the Zn interface is reduced and insufficient to promote complete hydration of Zn ions. The generation of this low-coordination solvation structure enhances the reaction kinetics of the desolvation process of Zn ions, suppresses the HER process, and promotes the uniform deposition of Zn ions. To investigate the desolvation kinetics of Zn^2^⁺, Nyquist plots of Zn|Zn symmetric cells using both the BE and the BE + SNC additive were measured at different temperatures to determine the activation energy (Ea) for desolvation. The results show that the Ea decreases from 66.3 kJ mol^−1^ (in BE) to 61.8 kJ mol^−1^ upon the formation of an SNC-based liquid/solid hybrid interfacial layer. This reduction in Ea suggests that the introduction of an unsaturated solvation structure facilitated by SNC effectively lowers the energy barrier for Zn^2^⁺ desolvation (Fig. S7).

To confirm this, HER was visualized via in situ optical microscope observation in an electrochemical cell. Zn dendrites grew more aggressively, and bubbles happened obviously in the aqueous BE environment (Fig. 1f, g). In comparison, no significant H_2_ evolution and more smooth Zn plating were found with BE + SNC electrolyte under the same current density of 10 mA cm^−2^. This uneven Zn deposition and H_2_ evolution is attributed to the long-range organized H_2_O−H_2_O hydrogen bond network and eventually accelerates parasitic reactions. To quantitatively determine the amount of hydrogen released, in situ electrochemical gas chromatography (EC-GC) was applied to monitor the H_2_ concentration during the Zn deposition. In BE, it is evident that HER is more severe with large bubbles due to the desolvation process of H_2_O happening at the Zn anode surface and the high area surface ratio of Zn dendrites in Fig. 1e. The Zn anode in BE + SNC released lower amounts of H_2_ than that of the Zn anode in BE, indicating the obvious H_2_ suppression in BE + SNC (Figs. 1e and S8). Subsequently, H_2_ evolution in BE exhibited almost 4.4 times higher than that of the Zn anode in BE + SNC. In situ quantitative testing of H_2_ evolution provides the most direct and powerful evidence that SNC has a "volume effect" in the liquid/solid mixed layer to inhibit HER and improved reversibility of Zn anodes during the Zn plating process, which is consistent with the results of in situ optical microscope observation (Fig. 1h).

### Analysis of Zn Anode Interface Behavior

Firstly, XPS and FTIR were employed to verify the absorption ability of the SNC on the Zn anode surface. In the XPS spectra (Fig. 2a), the characteristic peaks of C 1* s* located at 284.8, 286.3, and 288.9 eV could be assigned to C–C/C–H, C=O, and C–O bonds, respectively [26, 27]. The intensity of C=O and C–O peaks was increased in BE + SNC, evidencing the adsorption of SNC on the Zn anode surface cycled under 1 mA cm^−2^ and 1 mAh cm^−2^. This SNC absorption also enhances the hydrophilicity of the Zn surface, which is also verified by contact angle (CA) measurement. The BE + SNC improved the electrolyte-wetting ability of the Zn anode, which is reflected by enhanced interfacial contact compared with the BE (Fig. S9). To further verify it in FTIR, the blueshifts of characteristic peaks located at 1162 cm^−1^ (S=O vibration) and 896 cm^−1^ (symmetric C–O–S vibration) correspond to the sulfate group are likely attributed to the interaction between metallic Zn and SNC molecules (Fig. S10a) [28].

Moreover, as shown in Fig. 2b, when absorbed on Zn anode surface, SNC molecules possessed a higher position of the highest occupied molecular orbital (HOMO) than H_2_O molecules (− 1.044 vs. − 8.471 eV). This implies that SNC molecules are more likely to lose electrons as sacrificial agents for the H_2_O, reducing the propensity for HER on the surface of Zn metal [29]. On the other hand, compared with the H_2_O molecules (− 0.25 eV), the SNC molecule with sulfate groups has higher adsorption energy with the Zn anode (− 1.73 eV), which indicates that the H_2_O molecules on the anode–electrolyte interface were replaced by SNC molecules (Fig. 2c). The preferential adsorption of SNC at the electrode–electrolyte interface provided a mechanism for modulating interfacial Zn^2+^ coordination. Furthermore, as shown in dual descriptor iso-surfaces (blue lobes represent nucleophilicity), nucleophilic sites are concentrated on the OSO_3_^−^ of SNC, which is consistent with the results of FTIR (Fig. 2d) [30]. These observations provide strong evidence for the building of liquid/solid hybrid layer in electrolyte/anode interface by promoting SNC uniform adsorption on the Zn metal anode surface due to the strong adsorption capacity at its nucleation site OSO_3_^−^. The carbon chain of SNC is hydrophobic; hence, the grafted OSO_3_^−^ on SNC rarely fully coordinates with H_2_O compared to the free anion, which almost coordinates with H_2_O (Fig. S4b). As a result, in the BE + SNC electrolyte, the content of H_2_O molecules is relatively low due to the limited coordination ability between OSO_3_^−^ from SNC and H_2_O. The peak area of v_s_(O–H) at 1631 cm^−1^ was integrated to estimate the interfacial water content (Fig. 2e). The integral information of water peaks at different potentials shows that the addition of SNC reduces the enrichment of interfacial H_2_O.

In in situ EIS measurements, the Zn anode exhibits stable interfacial impedance during continuous cycling in the BE + SNC electrolyte, benefiting from reduced interfacial H₂O coverage and a higher HOMO level. This minimal change in impedance reflects the formation of a stable and robust interfacial layer with suppressed side reactions. The limited generation of by-products, including those from the HER, prevents disruption of the interface and facilitates uniform Zn deposition, further confirming the enhanced interfacial stability (Fig. 2f, g). In conclusion, SNC hinders the active water molecules being brought to the electrolyte/anode interface during the desolvation process and releases more free water, thereby inhibiting the decomposition of active water at the electrolyte/electrode interface such as H_2_ evolution. From electrochemical test results, Zn|Zn symmetric cells using the BE + SNC electrolyte exhibited slightly higher nucleation overpotential (75.3 mV) than pure ZnSO_4_ electrolyte (62 mV). The larger the nucleation overpotential contributes to the smaller nuclear radius, indicating that SNC facilitates uniform deposition (Fig. S11).

Time-of-flight secondary ion mass spectrometry (ToF–SIMS) was used to investigate by-productions and distribution of elements on the Zn anode surface after the Zn deposition process. It can be clearly seen that the O element is highly corrosive to the Zn anode without the addition of SNC (Fig. 2h, i). However, the Zn anode cycled with the introduction of SNC exhibited thinly O element distributed on the surface of the Zn anode, indicating that the SNC significantly suppressed passivation due to the formation of HER by-products [31]. In conventional desolvation processes, the occurrence of side production suppressed Zn-ion transport leading to high surface resistance after cycling. Based on the Zn anode surface analysis, the new interface solvation structure has significantly suppressed the side reaction in the Zn anode surface and enhanced the uniform Zn deposition.

### Zn Metal Anode with the Modification of SNC

**Fig. 3:**
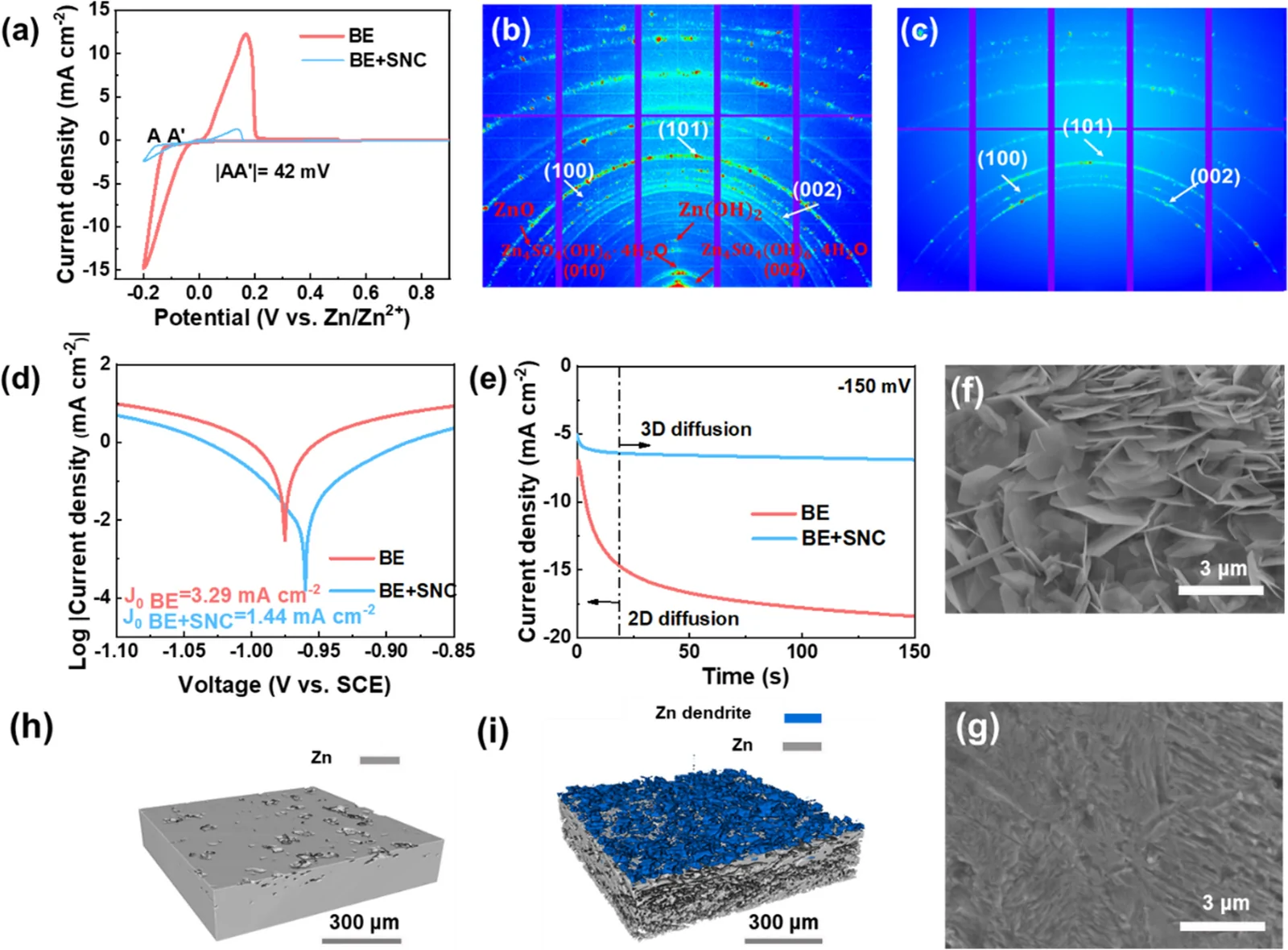
**a** Cyclic voltammetry (CV) curve of Zn|Cu asymmetric cell tested with/without SNC; 2D GIXRD image for Zn anode surface cycled in **b** BE; **c** BE + SNC; **d** Tafel plots of the Zn anode tested in BE and BE + SNC at a scan rate of 1 mV s^−1^; **e** chronoamperometry test for Zn|Zn symmetric cell with/without SNC; SEM of Zn anode surface **f** without SNC; **g** with SNC; CT of Zn anode surface cycled **h** with SNC; **i** without SNC

With the addition of SNC, the deposition exhibits a more stable deposition behavior. In FTIR, the blueshifts of *v*(SO_4_^2−^) occurred with the increasing content of SNC, indicating that causes more SO_4_^2−^ ions to dissociate at the interface, alleviating the cation loss near the anode surface and inhibiting random dendrite growth (Fig. S10b). Cyclic voltammetry (CV) was conducted to further evaluate the interfacial reaction kinetics by analyzing the initial Zn plating behavior (Fig. 3a). Compared to the BE, the BE + SNC exhibited a lower peak current density and a higher nucleation overpotential of 42 mV in Zn|Cu asymmetric cell. This increase in nucleation overpotential and reduced peak suggests a delayed nucleation process, typically associated with more homogeneous Zn^2^⁺ deposition with fine grain size, which is consistent with the in situ EIS measurements. Overall, a more stable and smooth interfacial layer constructed by SNC promotes uniform Zn deposition and enhances interfacial electrochemical stability [32].

The corrosion for the Zn anode is mainly attributed to the HER. Since the production of by-products can be directly linked to HER, analysis of by-products can indirectly assess the severity of HER. To clearly verify the side reactions and by-products on the Zn anode surface, 2D synchrotron grazing-incidence X-ray diffraction (GIXRD) patterns were recorded to analyze the effects of SNC on the texture control of Zn deposition. As shown in Fig. 3b, c, after 100 stripping/plating cycles, Zn foils cycled using BE exhibited peaks at 2θ angles of 6.23° and 8.93°, indexing to the Zn (002) and (010) planes of Zn_4_SO_4_(OH)_6_∙4H_2_O by-products (PDF#44–0673), respectively. This phenomenon indicates that significant corrosion reactions occurred on the anode surface during the plating/stripping process [33, 34]. Moreover, due to severe side reactions, electrochemically inactive Zn(OH)_2_ and ZnO species also existed in Zn foils cycled by BE [35]. These non-conductive and inert products ineffectively consume metallic zinc and produce “dead Zn.” However, with the addition of SNC, these inactive species were significantly reduced, demonstrating the corrosion mitigation and side reaction suppression capabilities of the SNC additive.

The optimized water-deficient electrolyte/anode interface also inhibits corrosion at the Zn metal surface and improves the corrosion resistance of the Zn metal anode. In the Tafel plot, with the addition of the SNC, after reducing the presence of H_2_O molecules at the interface, the corrosion current is significantly reduced (Fig. 3d). The BE + SNC electrolyte exhibited positive-shift potentials and lower exchange current densities (1.44 vs. 3.29 mA cm^−2^) than pure ZnSO_4_, confirming that the SNC improved the corrosion resistance of the zinc anode. Hence, according to the chronoamperometry (CA) results (under a constant overpotential of − 150 mV), the current density decreased rapidly in the first 20 s without the assistance of SNC, suggesting that the absorbed Zn ions diffused laterally on the Zn anode surface (Fig. 3e) [36]. By contrast, the SNC-modified electrolyte showed stable and low current response after 20 s, associated with the constrained 2D diffusion and uniform Zn deposition [37].

Furthermore, the electrodeposition morphologies and cross-sectional views of the Zn anode were observed by scanning electron microscopy (SEM) and synchrotron computed tomography (CT) measurements to confirm the inhibition effect of the BE + SNC on Zn dendrite growth (Fig. 3f, g). In BE, large and aggregated lamellar dendrites were observed on the Zn anode after stripping/plating for 100 cycles at a current density of 1 mA cm^−2^. In contrast, the surface of the Zn anode remained smooth when using the SNC. The CT technique was utilized to comprehensively observe the Zn anode surface after cycling (Fig. 3h and i), which is consistent with SEM results [38]. Cross-sectional analysis reveals pronounced dendrite growth in the BE sample, whereas such stacked dendritic structures are nearly absent in the BE + SNC system, indicating significantly improved Zn deposition behavior with the addition of SNC (Fig. S12). The porous structure is mainly produced by the accumulation of HER and alkali products, which is suppressed by the addition of SNC [38]. Both top-view and side-view images verified irregular and loosely porous zinc deposited in the BE, whereas SNC induced dense and uniform zinc deposition (Figs. S13 and S14). Furthermore, in order to evaluate the long-term cycle stability of the Zn anode, we also applied SEM to see the cross-section of Zn anode after cycle 200 cycles (Fig. S15). In the view of the SEM, it can be seen that without the addition of SNC, Zn deposition exhibited non-uniform morphology and partial infiltration into the glass fiber separator, indicating uncontrolled growth and poor deposition selectivity. This non-uniform deposition is primarily attributed to interfacial water-induced side reactions, vigorous parasitic processes, and the loose packing of zinc deposits. On the contrary, due to the participation of SNC, its deposition presents a uniform deposition pattern. Few side reactions promoted the deposition of Zn in a dense form and uniformly on the surface of Zn metal (Fig. S15). As a result, it is evident that the addition of SNC prevented the formation of a porous structure on the Zn anode surface.

### High Reversibility of Zn/Zn^2+^ After the Zn Deposition Process

**Fig. 4:**
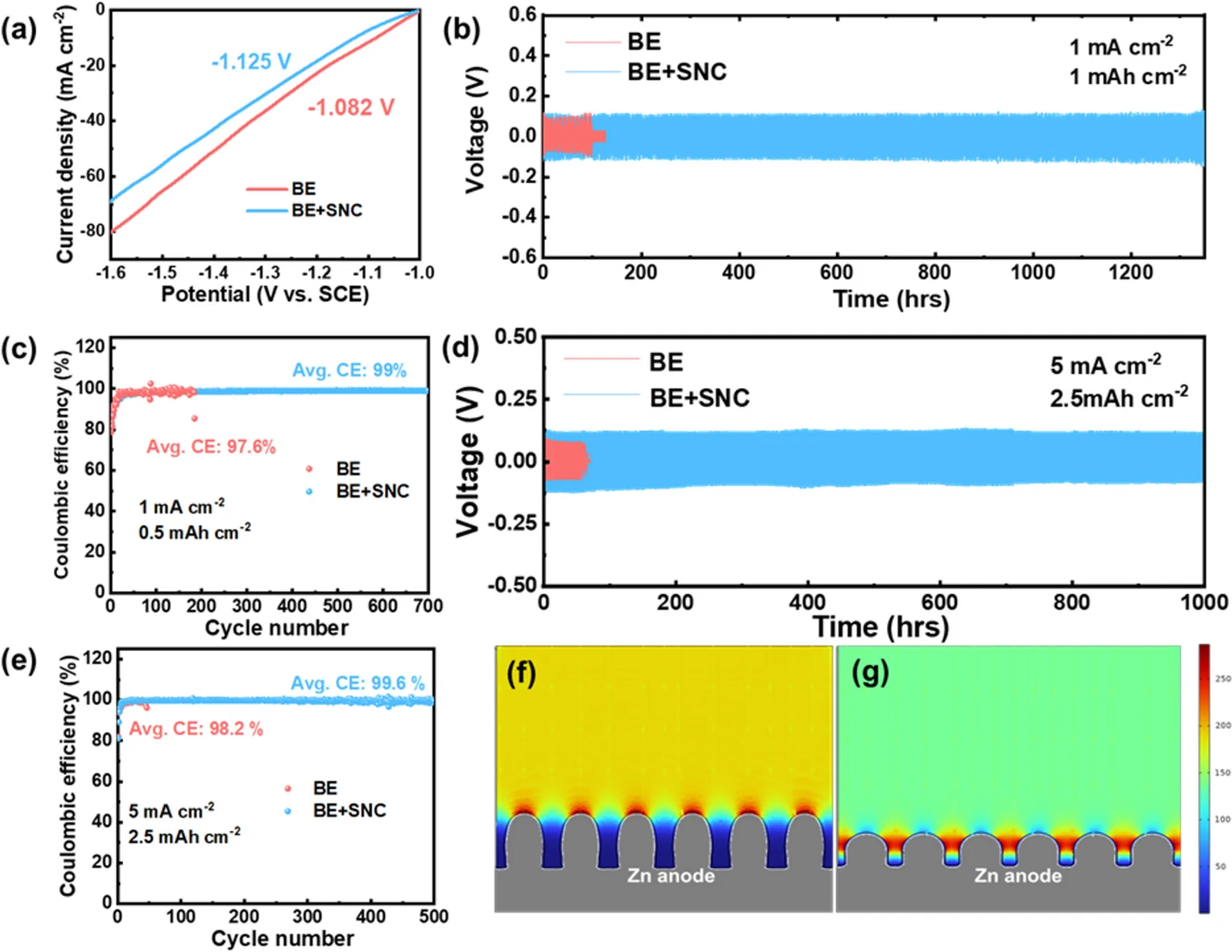
**a** Linear sweep voltammetry (LSV) of BE and BE + SNC; **b** Zn|Zn symmetric cell cycled at condition of 1 mA cm^−2^ and 1 mAh cm^−2^; **c** CE of Zn|Ti cell at condition of 1 mA cm^−2^ and 0.5 mAh cm^−2^ with/without SNC; **d** Zn|Zn symmetric cell cycled at condition of 5 mA cm^−2^ and 2.5 mAh cm^−2^; **e** CE of Zn|Ti cell at condition of 5 mA cm^−2^ and 2.5 mAh cm^−2^ with/without SNC; electric field distribution in the Zn anode surface **f** in BE; **g** in BE + SNC

Furthermore, the BE + SNC electrolyte reduced H_2_O to contact with the Zn anode and significantly expanded the electrochemical stability window, suppressing water-induced H_2_ evolution (Fig. 4a) [39]. In contrast to the significant current response of − 1.082 V vs. SCE, the current response shifted negatively to − 1.125 V upon adding the SNC additive, indicating that hydrogen evolution from SNC-containing electrolytes is suppressed compared to BE. Based on these, the stability and reversibility of the Zn anode in different electrolytes were evaluated. Under low current density at 0.5 mA cm^−2^ and a capacity of 0.5 mAh cm^−2^, the Zn|Zn symmetric cell with the BE + SNC electrolyte showed superb cycling stability for over 2000 h, which in the BE the stability is just 50 h (Fig. S16a, b). The Zn|Zn symmetric cell with the ZnSO_4_-SNC electrolyte showed superb cycling stability for over 1346 h at a current density of 1 mA cm^−2^ and a capacity of 1 mAh cm^−2^ (Fig. 4b). In contrast, cells using BE were short-circuited after 108 h in the same conditions. More attractively, even at a higher current density of 5 mA cm^−2^ (a capacity of 2.5 mAh cm^−2^), the Zn|Zn symmetric cell exhibited a promising lifespan of 1000 h (Fig. 4d), which was superior to the cell using BE (around 65 h) and outperformed most previous works (Fig. S17) [40–48]. With the addition of SNC, the Zn|Ti cell can maintain at least 99% CE for 700 cycles, which is much higher than without SNC (97.6%, 185 cycles) (Figs. 4c and S18). Furthermore, in the condition of 5 mA cm^−2^ and 2.5 mAh cm^−2^, the CE with the addition of SNC has significantly improved (99.6%, 500 cycles), which is 98.2% for 50 cycles without the addition of SNC (Fig. 4e). Moreover, as displayed in Fig. S19, based on the current density and cumulative plated capacity (CPC), a rational evaluation model of CE was employed to compare our work with other reported literature [49]. The BE + SNC exhibited a high CPC of 800 mAh cm^−2^ at 5 mA cm^−2^. Under the high current density cycling condition, the “Tip effect” has been enhanced, which makes Zn more accessible to form dendrites in Zn deposition. These results are verified by the FEA simulation (Fig. 4f, g). Once Zn^2+^ is deposited on metallic Zn unevenly, it forms bulges on the surface of the Zn anode. During continuous deposition, the electric field distribution on the surface of metallic Zn becomes increasingly uneven. More electrons tend to gather at the top of the bulges, forming "dead Zn" due to the "tip effect," particularly at high discharging rates. Comparatively, the Zn anode surface showed uniform electric field distribution in the BE + SNC electrolyte.

### High Reversibility of Zn/Zn^2+^ after the Zn Deposition Process

**Fig. 5:**
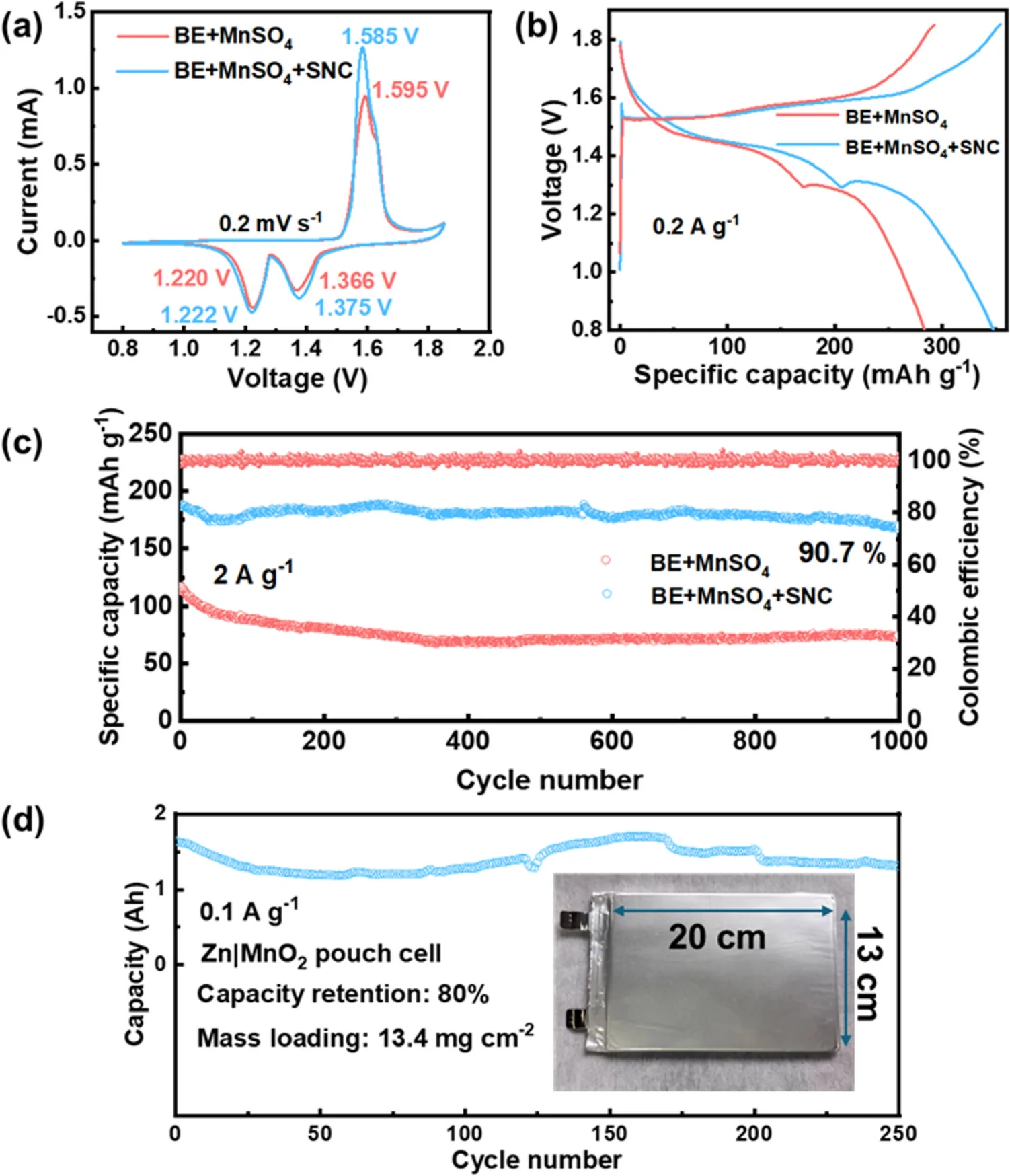
**a** CV test for Zn|MnO_2_/CNT full cell; **b** GCD curves for Zn||MnO_2_/CNT full cell with/without SNC; **c** Zn|MnO_2_ full cell at condition of 2 A g^−1^ with/without addition of SNC; **d** Zn|MnO_2_ pouch cell with 0.1 A g.^−1^ and 1.5 Ah capacity (inset: photograph of stacked Zn|MnO_2_ pouch cell)

To evaluate the role of SNC additives in the reversible capacity of full cells, Zn|MnO_2_/CNT and Zn|SNC|MnO_2_/CNT batteries were assembled. XRD analysis confirmed that the synthesized MnO_2_/CNT cathode material exhibits the characteristic peaks of α-MnO_2_ (JCPDS No. 44–0141), with preferential growth along the (110) plane, indicating typical nanocrystalline features and success synthesis of α-MnO_2_ (Fig. S20) [50, 51]. To evaluate the impact of the cathode in the system, cross-sectional SEM analysis was conducted on samples after 200 cycles. The MnO_2_/CNT cathode in the BE exhibited slight structural cracking, whereas the cathode in the SNC-containing electrolyte displayed a more uniform and intact morphology. This improvement is attributed to the enhanced Zn^2^⁺ ion transport kinetics facilitated by the SNC additive (Fig. S21). It should be noted that 0.1 M MnSO_4_ was pre-added into the BE and BE + SNC electrolyte to prohibit Mn dissolution and give rise to the reversible capacity in the full cell [52]. Except for similar Mn-ion redox peaks and Zn storage/delivery behaviors, the electrolyte with SNC additive realized distinct cathodic/anodic peaks shift to more positive/negative voltages and higher current densities, indicating lower interface impedance, raid electrochemical kinetics with better desolvation kinetics at interfaces (Fig. 5a) [53]. The Mn 2*p* XPS spectra show no noticeable peak shifts before and after cycling, with peaks at 654 and 642.2 eV corresponding to Mn 2*p*_1/2_ and Mn 2*p*_3/2_ [29]. The 11.8 eV separation confirms the dominant Mn^4+^ state, indicating high cathode reversibility in the BE + SNC electrolyte (Fig. S22) [54]. As a result, in Fig. 5b, the galvanostatic charge–discharge (GCD) test of Zn|MnO_2_/CNT battery using the ZnSO_4_ + MnSO_4_ + SNC electrolyte delivered a higher capacity of 347.6 mAh g^−1^, compared to 283.4 mAh g^−1^ in BE + MnSO_4_. With the formation of the H_2_O buffer layer and the design of interface Zn solvation structure, desolvation kinetics has been significantly enhanced, which optimizes the electrochemical performance of Zn|Zn symmetric cell and Zn|MnO_2_/CNT battery. In addition, we applied EIS to evaluate the interface of the Zn|MnO_2_/CNT full battery, although BE + MnSO_4_ and BE + MnSO_4_ + SNC exhibited similar interfacial impedance at the initial stages of cycling, noticeable divergence emerged after 30 and 100 cycles. In the BE + MnSO_4_, continuous impedance growth was observed, primarily due to intensified interfacial side reactions during cycling, which deteriorated the electrode/electrolyte interface stability (Fig. S23). Finally, Figs. 5c and S24 show the long-term cycling stability of the Zn|MnO_2_/CNT battery in different electrolytes. The full cell using the BE + MnSO_4_ + SNC electrolyte maintained a steady CE of around 100% and possessed long-lasting stability (90.7% retention for over 1000 cycles) than those using the BE + MnSO_4_ electrolyte (63.7% retention for over 1000 cycles), further proving that SNC additives could improve the cycling stability of the Zn-ion full cell. Optimized desolvation kinetics significantly improved the high-rate electrochemical performance of the battery. The Zn|MnO_2_/CNT battery exhibited higher capacity and rate performance with SNC (Figs. S25 and S26). Besides, the AZIB using BE + MnSO_4_ + SNC electrolyte outperformed the other electrolyte additives in terms of specific capacity and cycling stability (Fig. S27) [26, 55–57]. In addition, to approach commercial application, we fabricated a Zn|MnO_2_ pouch cell with 13.4 mg cm^−2^ loading, which remained stable over 250 cycles (Fig. 5d). In this context, our “interfacial solvation structure engineering” approach represents a complement to traditional electrolyte design and provides a new direction for precise interface regulation in aqueous Zn-based energy storage systems.

## Conclusions

In this study, through in situ ATR-FTIR and FI-EXAFS, we revealed the study of interfacial Zn-ion solvation structure for the first time. We achieved the combination of interfacial detection technology and electrochemical electrolyte design at the spectral level. After adding SNC, the construction of a water-deficient layer at the zinc metal interface was achieved, realizing a zinc-ion solvation structure with low solvation coordination. Consequently, the introduction of SNC significantly improves the reversible Zn plating/stripping behaviors observed in both Zn|Zn symmetric cells and asymmetric Zn|Ti cells. These improvements are substantial, with an average Coulombic efficiency of 99.6% over 500 cycles, even at a high current density of 5 mA cm^−2^. Furthermore, SNC-induced pouch cells achieved over 250 cycles at 0.1 A g^−1^ with 1.5 Ah capacity for practical application. Our work provides new insight into controlling Zn-ion hydration by interfacial H_2_O content via interfacial solvation structure engineering.

## Supplementary Information

Below is the link to the electronic supplementary material.

Supplementary file1 (DOCX 2893 KB)
