# Supplementary material for: Achieving Ah-Level Zn–MnO2 Pouch Cells via Interfacial Solvation Structure Engineering
Source: Nanomicro Lett. 2026 Jan 2;18:124. doi: 10.1007/s40820-025-01935-6 (PMC12757502; doi:10.1007/s40820-025-01935-6)
Supplement: Supplementary file 1 — Supplementary file1 (DOCX 2893 KB) [file 40820_2025_1935_MOESM1_ESM.docx]

Supporting Information for

**Achieving Ah-Level Zn–MnO_2_ Pouch Cells via Interfacial Solvation Structure Engineering**

Jing Wei^1,2,4^^,#^, Lichao Tan^1,#^, Qianyi Ma^1,2^, Xintao Long^3^, Shibin Li^1^, Yu Shi^2^, Rui Gao^2^, Zijing Xu^2^, Dan Luo^3,*^, Jie Zhang^3,*^, Dagang Li^4^, Xin Wang^1,*^, AipingYu^2,*^, Zhongwei Chen^3,*^

^1^ Institute of Carbon Neutrality, Zhejiang Wanli University, Ningbo 315100, P. R. China

^2^ Department of Chemical Engineering, Waterloo Institute for Nanotechnology, University of Waterloo, Waterloo, N2L 3G1, Canada

^3^ Power Battery and Systems Research Center, State Key Laboratory of Catalysis, Dalian Institute of Chemical Physics, Chinese Academy of Sciences, Dalian 116023, P. R. China

^4^ College of Material Science and Engineering, Nanjing Forestry University, Nanjing 210037, P. R. China

^#^Jing Wei and Lichao Tan contributed equally to this work.

*Corresponding authors. E-mail: [luo.dan@dicp.ac.cn](mailto:luo.dan@dicp.ac.cn) (Dan Luo); [zhangjie21@dicp.ac.cn](mailto:zhangjie21@dicp.ac.cn) (Jie Zhang); [wangx@zwu.edu.cn](mailto:wangx@zwu.edu.cn) (Xin Wang); [aipingyu@uwaterloo.ca](mailto:aipingyu@uwaterloo.ca) (AipingYu); [zwchen@dicp.ac.cn](mailto:zwchen@dicp.ac.cn) (Zhongwei Chen)

**S1 Supplementary Text**

**S1.1 Theoretical calculations**

**Density functional theory (DFT) calculations.** All quantum chemical calculations were performed using DFT methods as implemented in the Gaussian 16 program package. The B3LYP functional was employed with the 6-311++G(d, p) basis set. Geometry optimizations were conducted in an aqueous environment using the SMD (Solvation Model based on Density) implicit solvent model. The final structures were determined by energy minimization without imposing any molecular symmetry constraints. The binding energy between Zn^2+^, SO4^2-^, H_2_O, and SNC was defined as the interaction among the different molecular fragments. The binding energy, Ebind, was calculated according to the following equation (S1):

E=E_total_-E_x_-E_y_ (S1)

where E_total_ is the structure total energy, E_x_ and E_y_ is the energy of different molecule fragments (X, Y =Zn^2+^, SO_4_^2-^, H_2_O and SNC) according to the different structure configurations.

**Molecular dynamics (MD) simulation.** The BE and BE+SNC aqueous solution was calculated using COMPASS Ⅲ force field and Forcite tool in MS 2020. BE+SNC aqueous solution contains 150 ZnSO_4_ molecules, 15 SNC molecules and 4180 H_2_O molecules in a rectangular box with a length of 50.86 Å× 50.86 Å× 50.86 Å. There are no SNC molecules in the BE, and the rest are consistent with the BE+SNC. All MD calculations were first performed in the NVT ensemble (T=298.0 K) and the NPT ensemble (P= 1 atm), with a time step of 1 ps, and simulation times of 500 ps and 1ns, respectively. The running time is sufficient to stabilize the energy and temperature of the system. After that, the motion trajectory of each atom in the system was collected for 500ps in the NPT ensemble (T=298.0 K, P= 1 atm) for subsequent analysis. During this period, the simulation trajectory was recorded every 5000 steps. The temperature was controlled by a Nose-Hoover thermostat. The Ewald scheme and atom-based cutoff method (radius of 12.5 Å) were used to treat electrostatic and van der Waals (vdW) interactions, respectively.

**Finite Element Analysis Method.** COMSOL Multiphysics with the tertiary current distribution and the deformed geometry interface models were used to perform the simulations. The simulation area has a height of 80 μm and a width of 90 μm. The mesh is chosen to be triangular or tetrahedron-based while using an increasing refinement toward the electrode bands. The ion concentration in the electrolyte is determined by the conservation of current and the conservation of ion mass, as shown in the following equation (S2):

$\frac{\text{∂}\text{c}_{\text{i}}}{\text{∂}\text{t}}\text{=}\text{-}\text{∇}\text{·}\text{N}_{\text{i}}\text{+}\text{R}_{\text{i}}$  (S2)

where, *c_i_* represents the concentration of the substance, *N_i_* represents the flux density of the substance, and *R_i_* is the source term generated by electrochemistry. The transport of Zn^2+^ can be described by the combination of migration and diffusion, as shown in Equation (S3)：

$\vec{N}_{i}=-D_{i}\nabla c_{i}-z_{i}c_{i}\mu_{i}F\nabla\phi_{e}$ (S3)

Where, *D_i_* represents the diffusion rate of substance *i*, *F* is the Faraday constant (96485 C/mol), *ϕ_e_* denotes the electrolyte potential, and *z* is the charge number of substance *i*. *μ_i_* is the electrical mobility, which is calculated using the following Nernest-Einstein equation (S4):

$\mu_{i}=D_{i}/RT$ (S4)

where, *R* is the gas constant（8.314 J/(mol·K)）and *T* is temperature. The cathodic reaction is defined by the Butler-Volmer equation. However, with the SNC adsorbed on the surface, the Butler-Volmer equation should take the surface concentration into consideration, which is given by equation (S5):

$i_{loc}=-(C`-k\theta)exp(\frac{-(\alpha-k`\theta)F\eta}{RT})i_{0}$ (S5)

where *θ* is the coverage of adsorbed inhibiting additive and cannot exceed unity, *C`* is the coefficient of Zn^2+^ concentration, *k* is coefficient of SNC concentration, *k`* is the inhibiting transfer coefficient of the SNC, *α* is the transfer coefficient of the cathode, *η* is the overpotential, *i_loc_* is the local current density, and *i_0_* is the exchange current density.

**S1.2 Electrochemical characterization**

In a typical process, MnO_2_/CNT composites were synthesized by a hydrothermal method. Firstly, 92.5 mM of Mn(CH3COO)2·4H2O was dissolved in 150 mL of DI water under stirring for 10 min. And then, 0.5 g MWCNT was added into the abovementioned solution slowly. Subsequently, the mixed solution was stirred for another 10 min before added into 115 mM KMnO_4_ aqueous solution. The mixture was continuously stirred for 30 min and then heated at 80 ℃ for 6 h. The products were washed with DI water for 1 h and collected by centrifugation. Then the brown MnO_2_/CNT products were obtained after being placed in a freeze-dryer for 12 h.

The CR2032-type coin cells with 120 μL electrolyte and glass fiber (Whatman, GF/D) as the separator were assembled for all Zn symmetric cells, Zn|Ti half cells and Zn-MnO_2_/CNT full cells in an open environment. For Zn-MnO_2_/CNT full cells, cathodes (Φ12 mm) were obtained by mixing the MnO_2_/CNT with super P and PVDF at a weight ratio of 7:2:1. Subsequently, the slurry was coated onto Ti foil and then dried at 80 °C for 12 h. The electrochemical windows of the aqueous electrolytes were tested by linear sweep voltammetry (LSV) at 5 mV s^−1^ in stainless steel (SS)|Zn cells. The corrosion, diffusion, and hydrogen evolution behaviors of Zn foil anodes were performed with a three-electrode system (Zn foil as working electrode, Pt as the counter electrode, and SCE as reference electrode) under VMP-300 electrochemical workstation. The Tafel plots were recorded at a scan rate of 1mV s^−1^ under a potential range of ±0.3 V versus open-circle potential of the system. The diffusion curves were recorded by chronoamperometry method under an overpotential of -150 mV. Nyquist plots of the symmetric Zn cell with BE and BE + SNC electrolyte at different temperatures to obtain desolvated activation energy E_a_. according to the Arrhenius equation (S6) as following:

$\begin{aligned} \frac{1}{R_{\text{ct}}}=A\exp(-\frac{E_{a}}{RT}) \end{aligned}$ (S6)

where *R*_ct_, *A*, *E*_a_, *R*, and *T* are the charge-transfer resistance, Arrhenius constant, desolvated activation energy, gas constant, and absolute temperature, respectively.

Galvanostatic charge and discharge tests of Zn-MnO_2_/CNT full cells were performed on LAND CT2001A battery system while CV tests were conducted by BioLogic VMP-300 electrochemical workstation.

# S2 Supplementary Figures


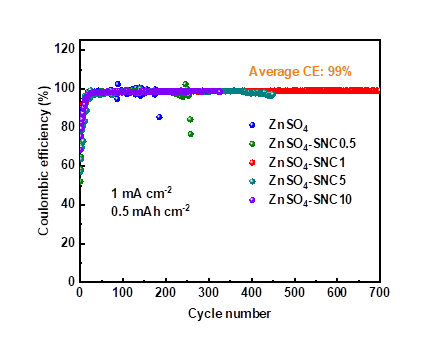


**Fig. S1** CEs of Zn plating/stripping without/with SNC at 1 mA cm^−2^ and capacity of 0.5 mAh cm^-2^


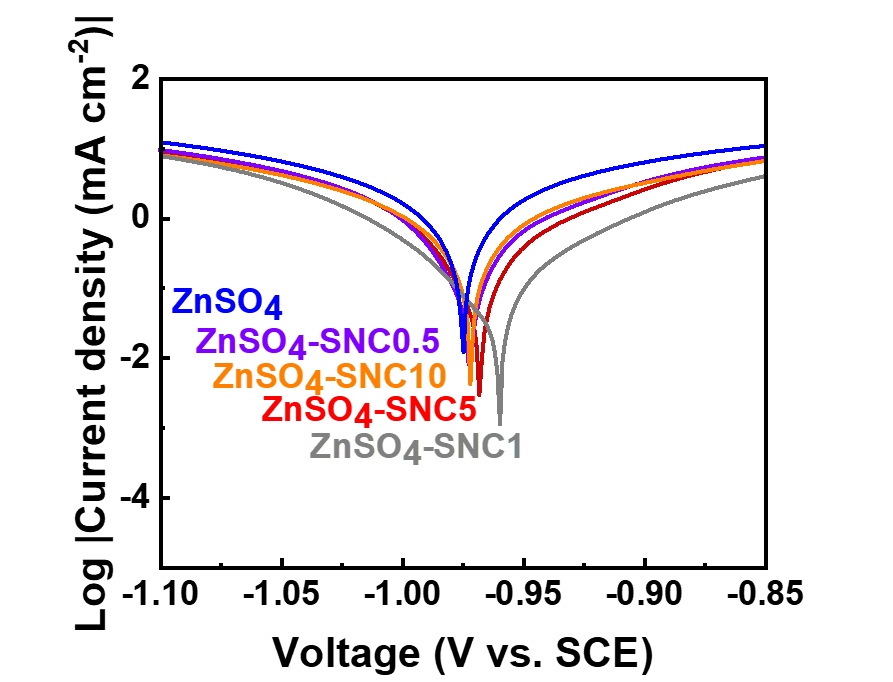


**Fig. S2** Tafel plots of the Zn anode tested with different SNC content at a scan rate of 1 mV s^-1^


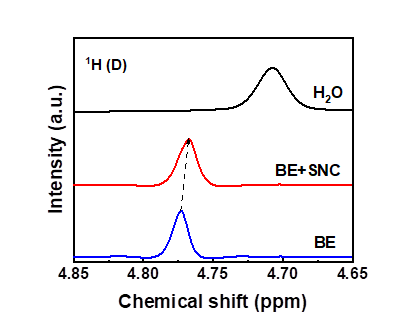


**Fig. S3** NMR for ^1^H in the BE and BE+SNC


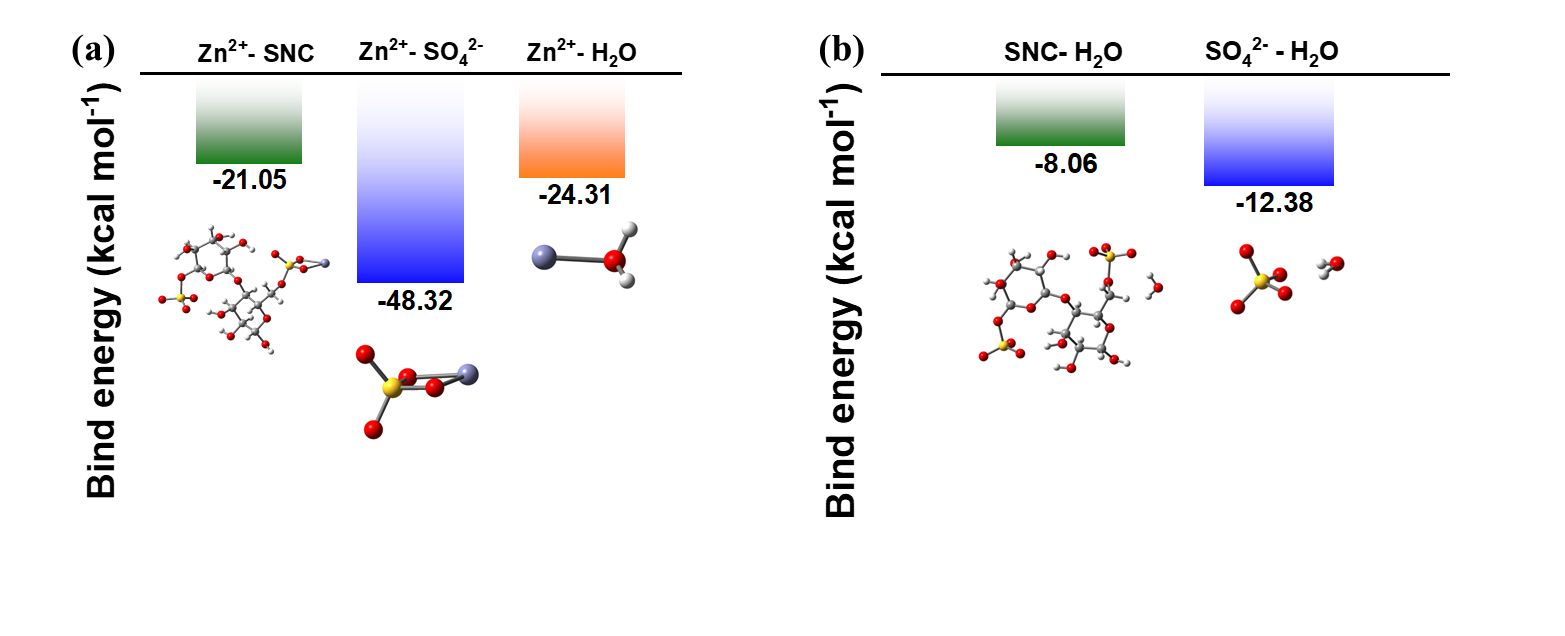


**Fig. S4** Binding energy between SNC and H_2_O, Zn^2+^ and SO_4_^2-^


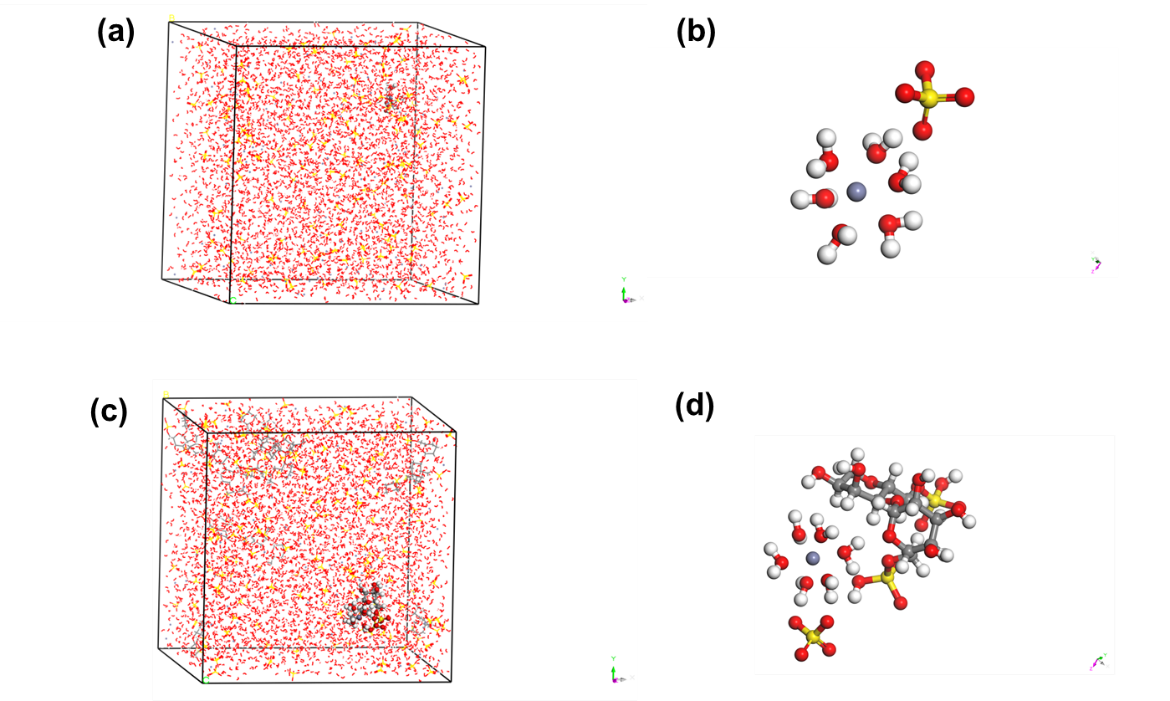


**Fig. S5** MD snapshot of (**a, b**) 2 M ZnSO_4_ aqueous electrolyte and enlarged corresponding solvation structure (**c, d**) SNC in 2M ZnSO_4_ aqueous electrolyte and enlarged corresponding solvation structure, respectively. Zn, S, O, C, and H atoms are represented by gray, yellow, red, gray, purple, and white balls, respectively


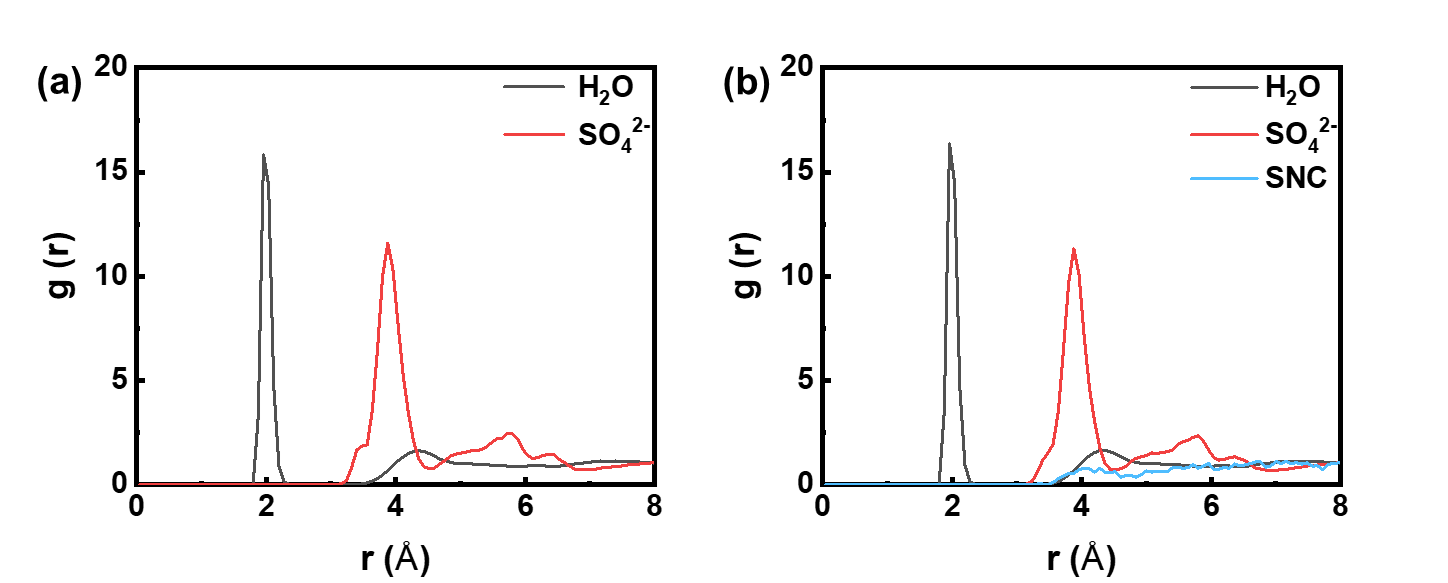


**Fig. S6** Radial distribution function (RDF) between Zn^2+^ and H_2_O, SO_4_^2-^, and SNC in (**a**) 2 M ZnSO_4_ aqueous electrolyte and (**b**) 2 M ZnSO_4_ aqueous electrolyte with SNC.


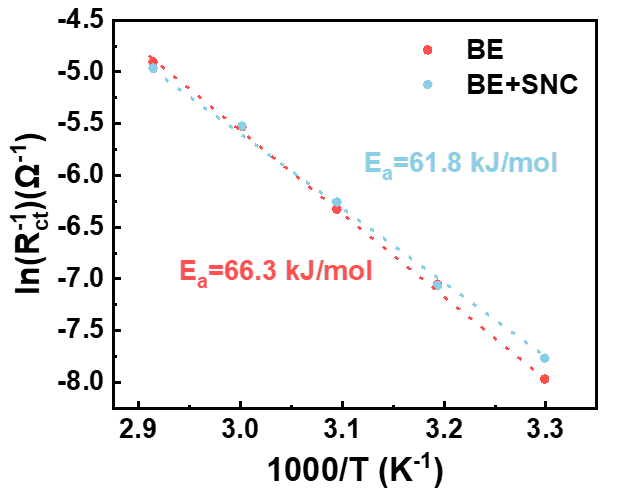


**Fig. S7** Nyquist plots of symmetric Zn cell with BE and BE + SNC electrolyte at different temperatures


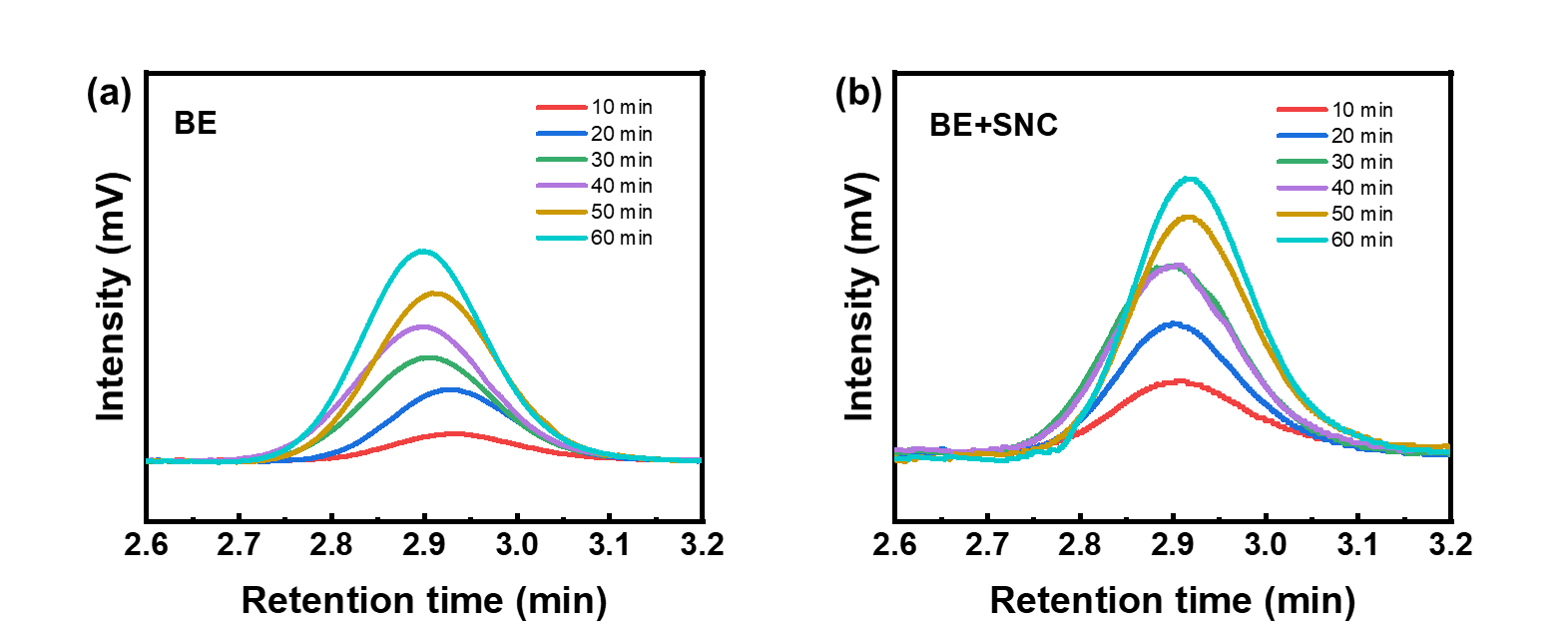


**Fig. S8** The in-situ EC-GC test of the Zn plating process on Zn metal in the electrolyte (**a**) without SNC and (**b**) with SNC

**
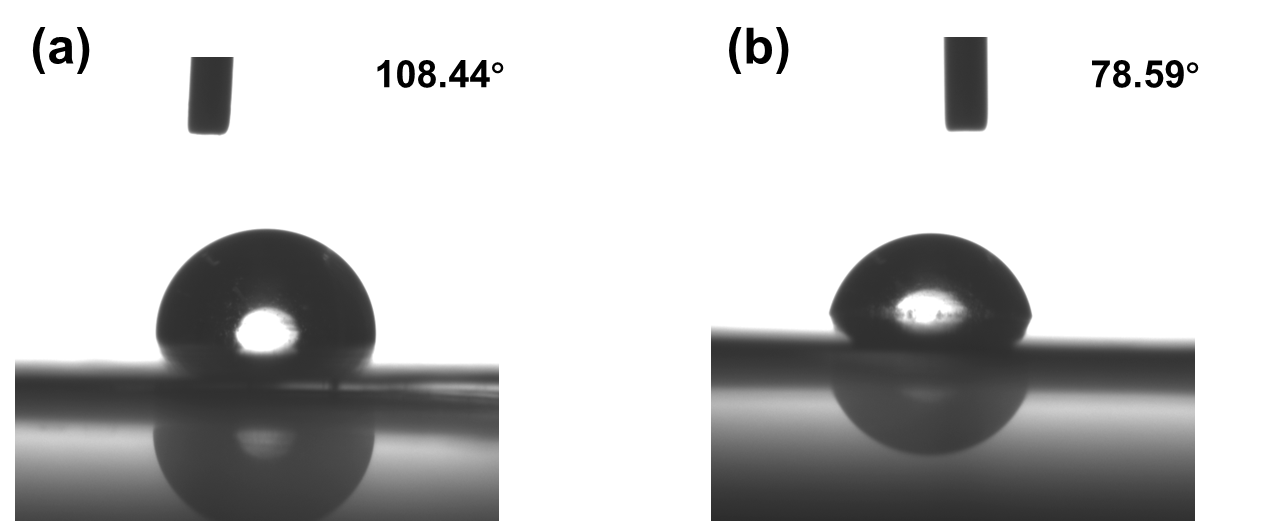
**

**Fig. S9** contract angle between Zn anode and electrolyte (**a**) without addition of SNC; (**b**) with the addition of SNC

**
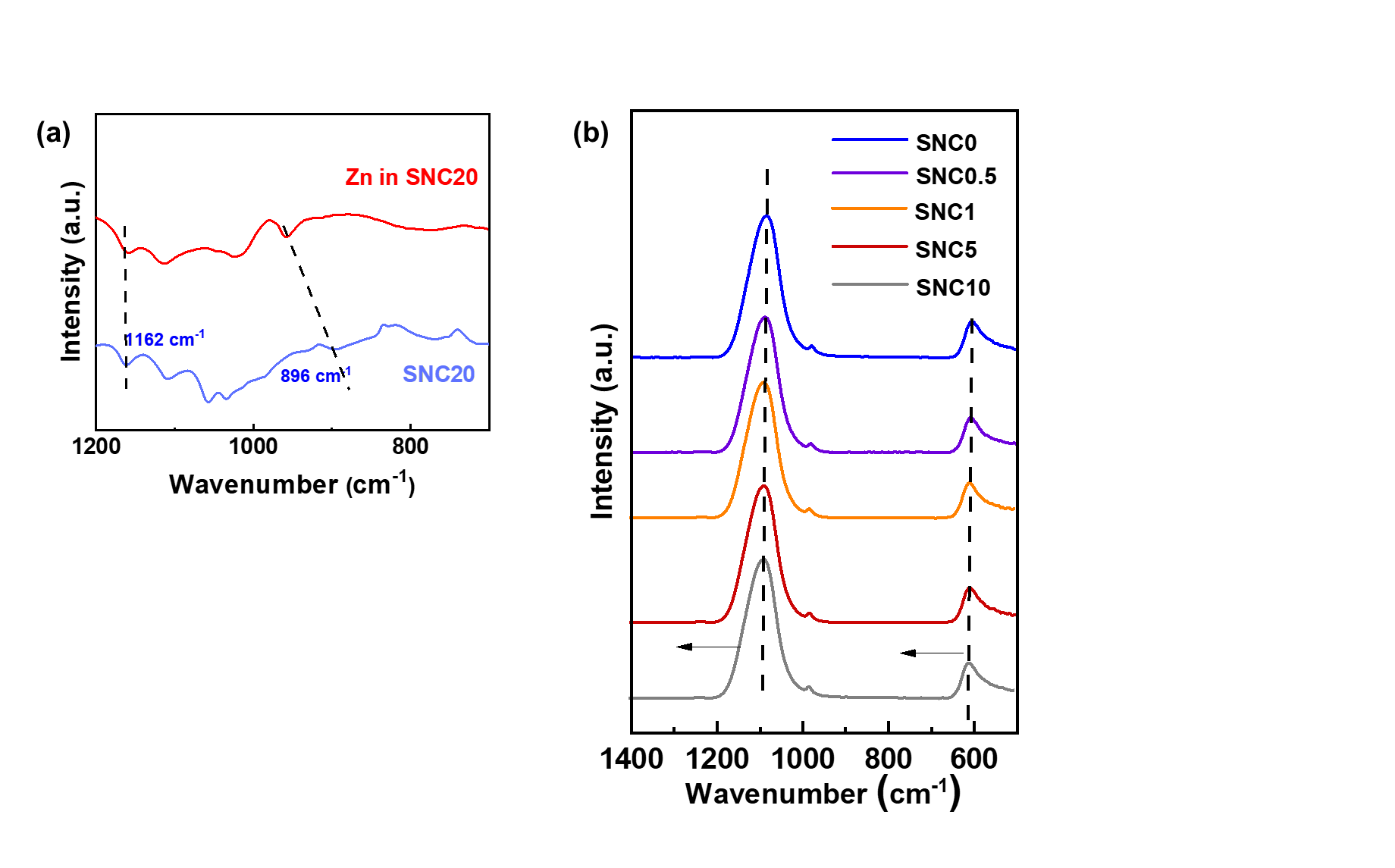
**

**Fig. S10** (**a**) FTIR of ZnSO_4_ electrolyte with the addition of different content of SNC; (**b**) FTIR of different content of SNC in the electrolyte


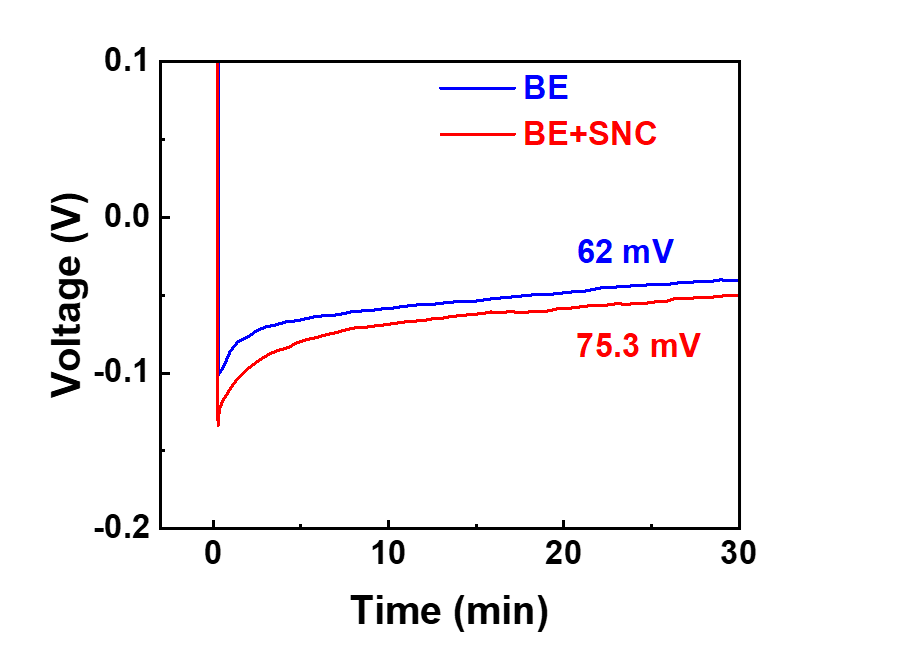


**Fig. S11** Overpotential of Zn deposition in BE and BE+SNC electrolyte, respectively


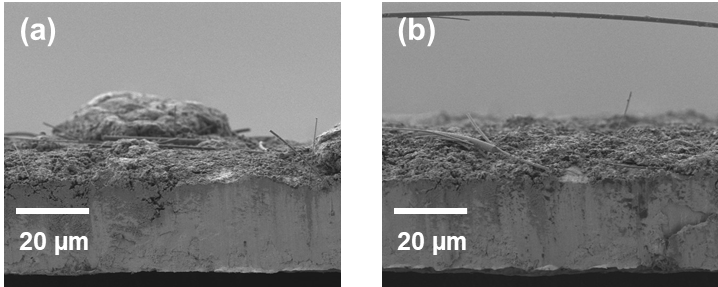


**Fig. S12** SEM of Zn anode surface (**a**) without SNC; (**b**) with SNC


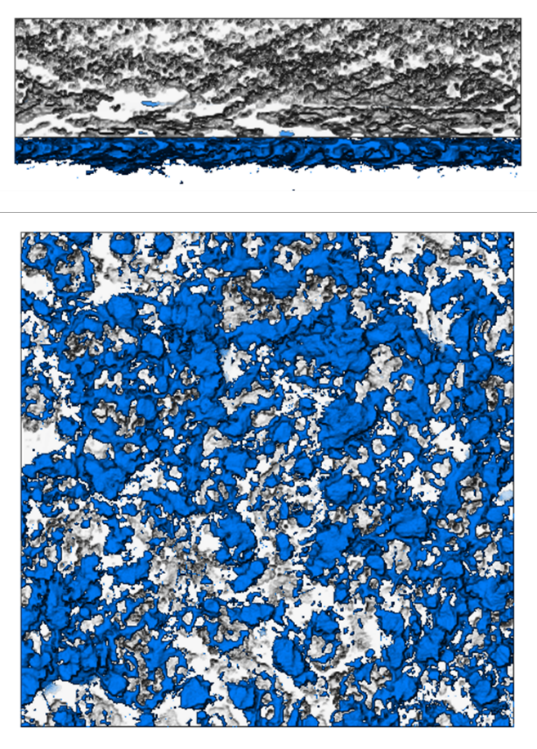


**Fig. S13** Top viewer and side viewer of CT of Zn anode cycled in the BE


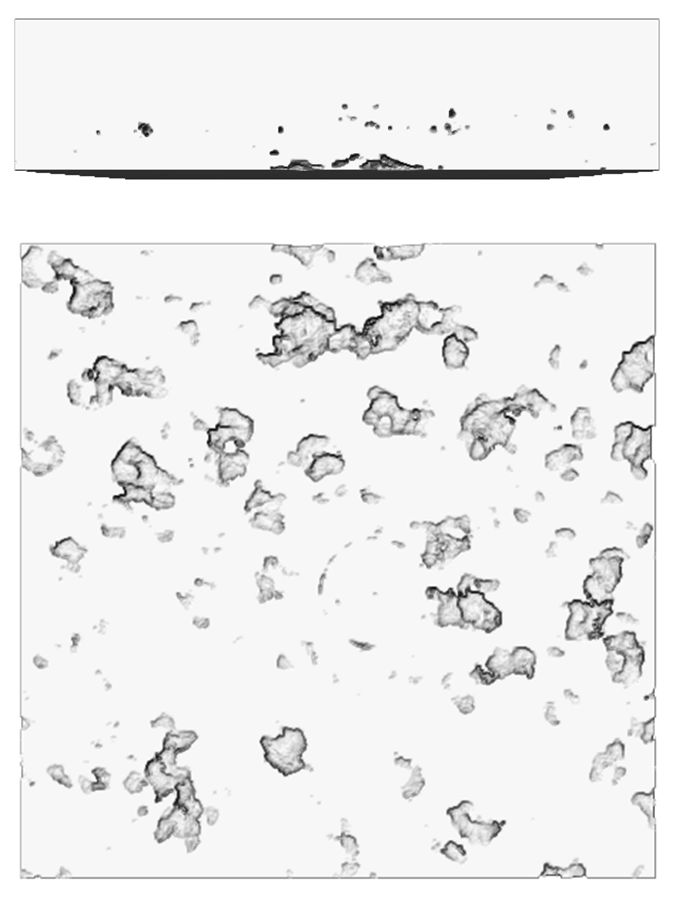


**Fig. S14** Top viewer and side viewer of CT of Zn anode cycled in the BE+SNC electrolyte


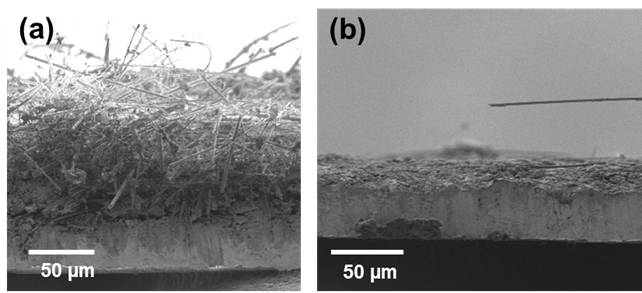


**Fig. S15** Cross-section SEM for Zn anode after long-term 200 cycles (**a**) BE; (**b**) BE + SNC


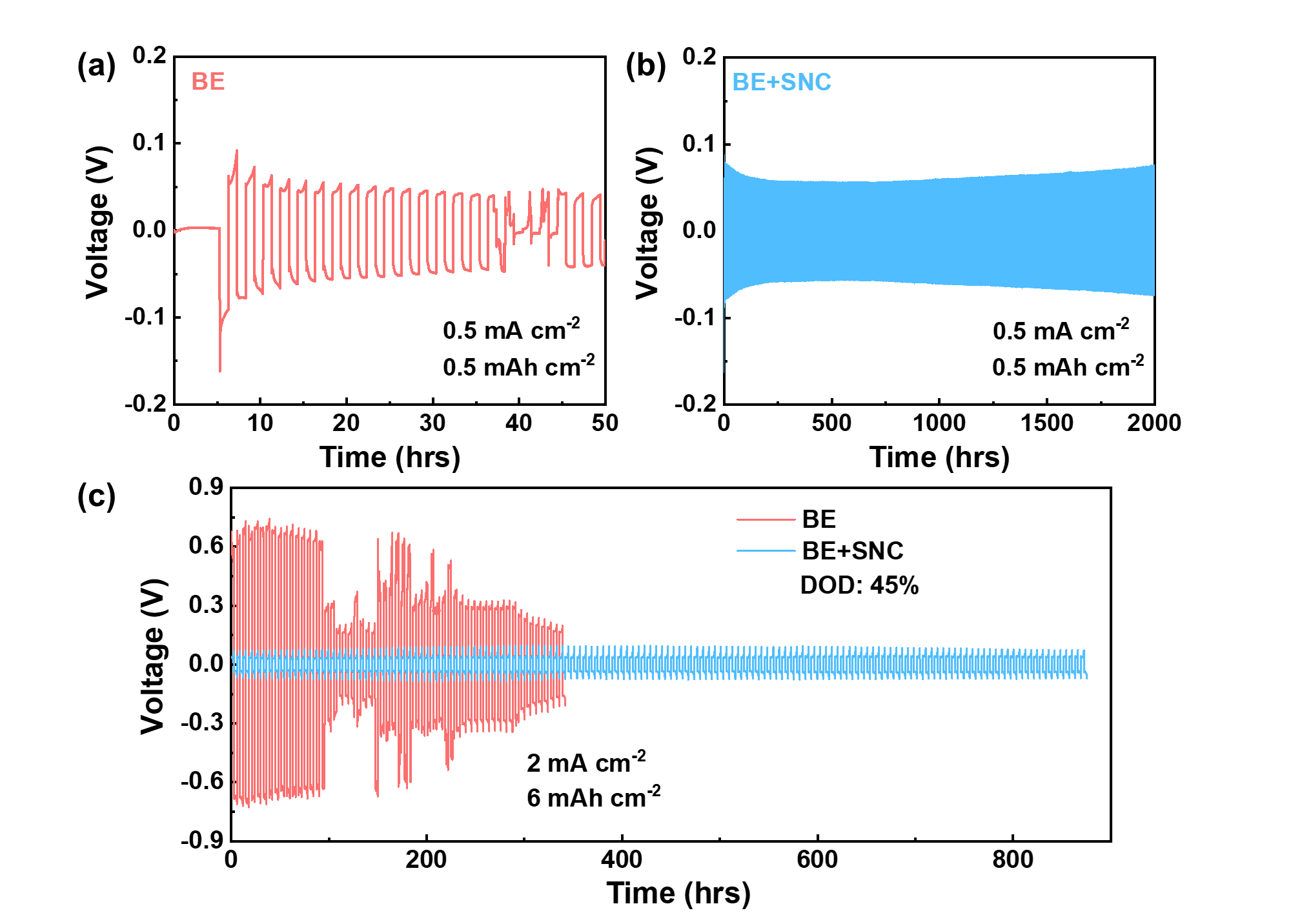
**Fig. S16** Zn|Zn symmetric cell cycled at condition of 0.5 mA cm^-2^ and 0.5 mAh cm^-2^ in (**a**) BE and (**b**) BE+SNC; (**c**) Zn|Zn symmetric cell cycled with a DOD of 45% at condition of 2 mA cm^-2^ and 6 mAh cm^-2^ in BE and BE+SNC, respectively


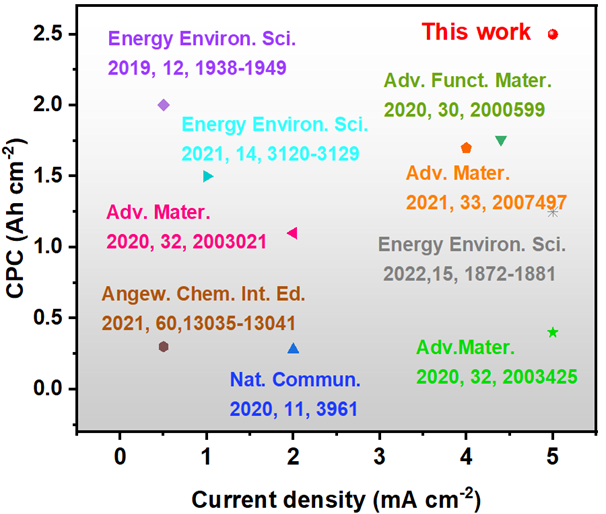


**Fig. S17** Zn|Zn symmetric cell with/without addition of SNC at condition of 5 mA cm^-2^/ 2.5 mAh cm^-2^


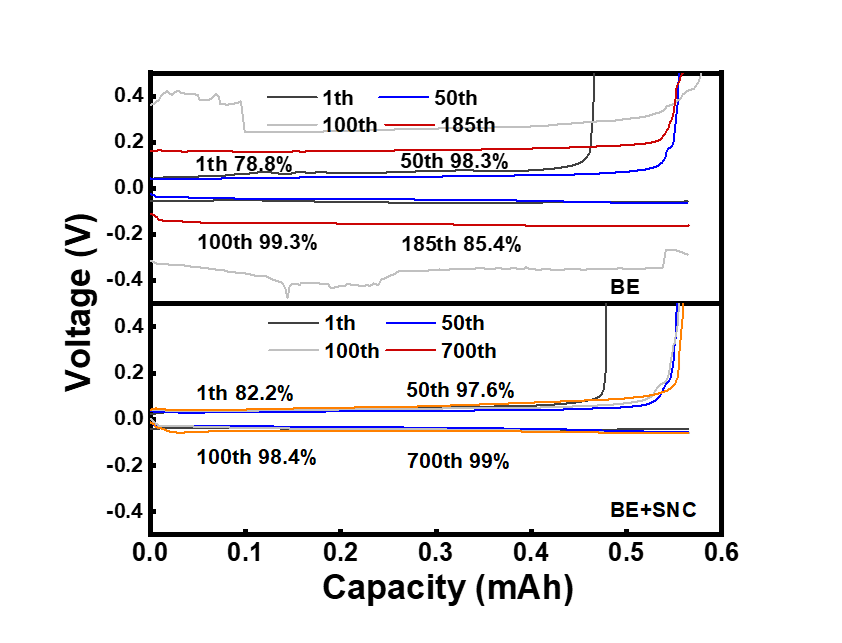


**Fig. S18** Zn plating/stripping profiles on Ti foil in BE (top) and BE+SNC (bottom) at 1 mA cm^-2^


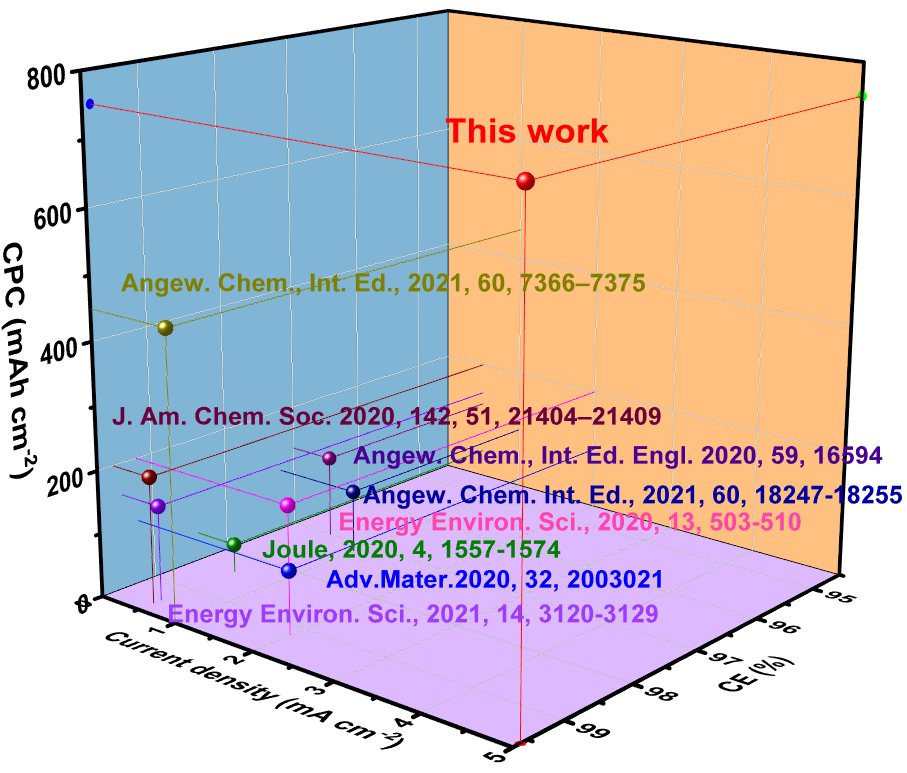


**Fig. S19** The comparison of the CPC and average CE with those of others reported literatures


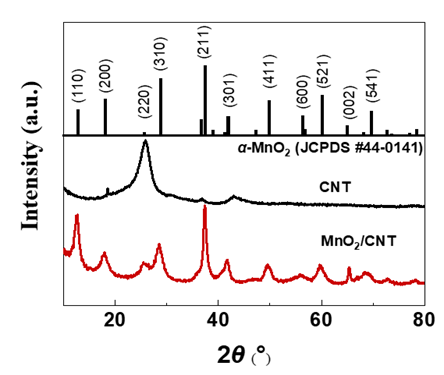


**Fig. 20** XRD patterns of the CNT and α-MnO_2_/CNT


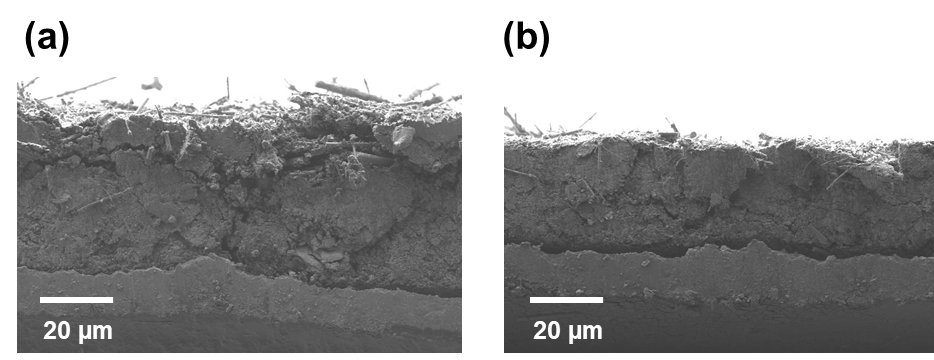


**Fig. S21** Cross-section SEM for MnO_2_/CNT cathode after long-term 200 cycles (**a**) BE; (**b**) BE + SNC


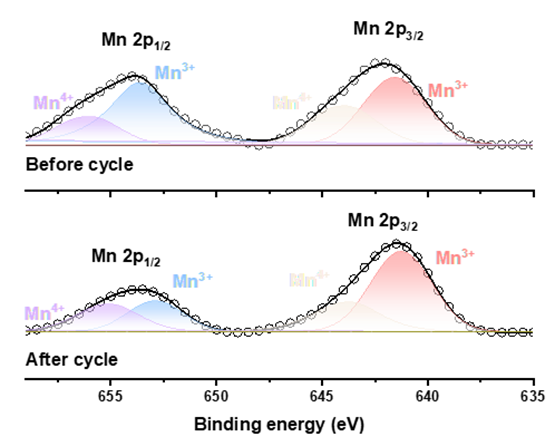


**Fig. S22** XPS for α-MnO_2_/CNT before cycling and after 30 cycles


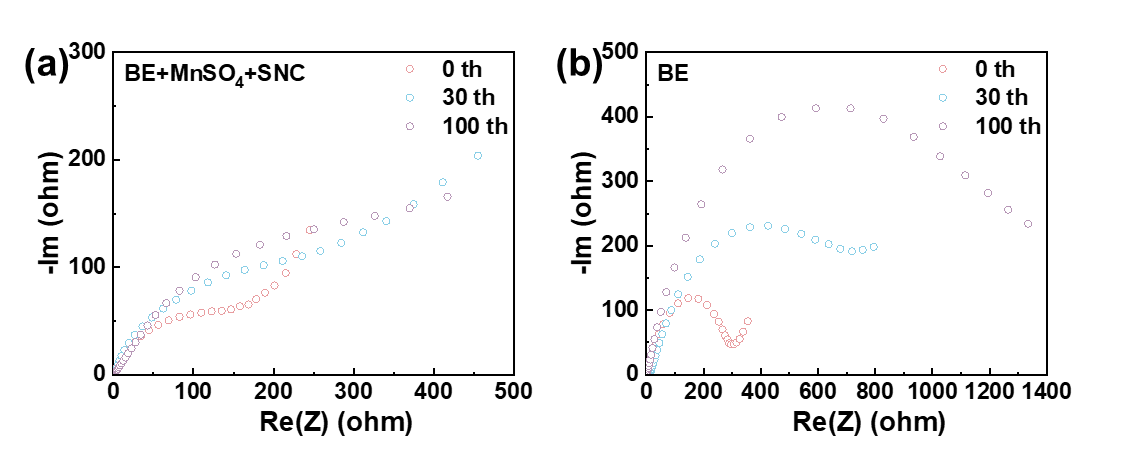


**Fig. S23** EIS plots for the Zn|MnO_2_/CNT battery after cycling 0, 30, 100 times


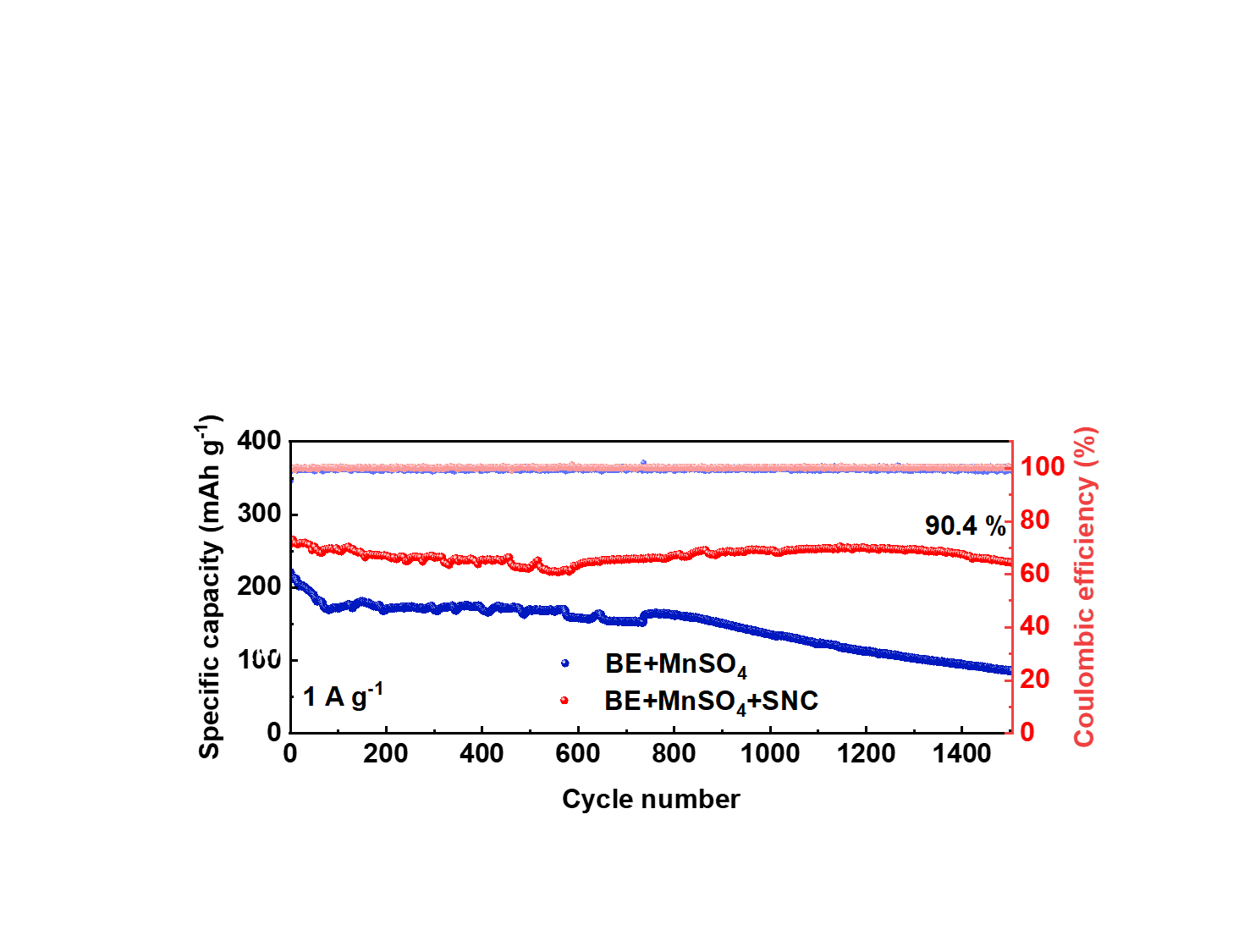


**Fig. S24** Long-term cycling stability of Zn-MnO_2_/CNT full cells in BE+MnSO_4_ and BE+MnSO_4_+SNC at 1 A g^-1^

**
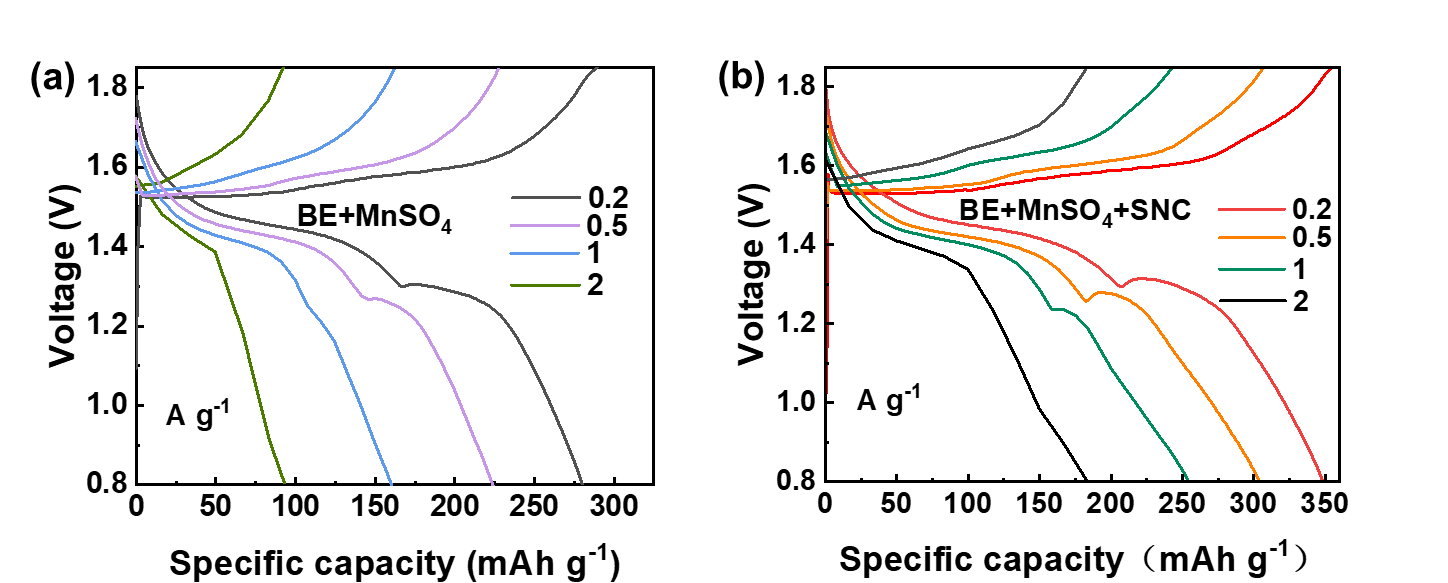
**

**Fig. S25** Charging/discharging curves in (**a**) BE+MnSO_4_ and (**b**) BE+MnSO_4_+SNC


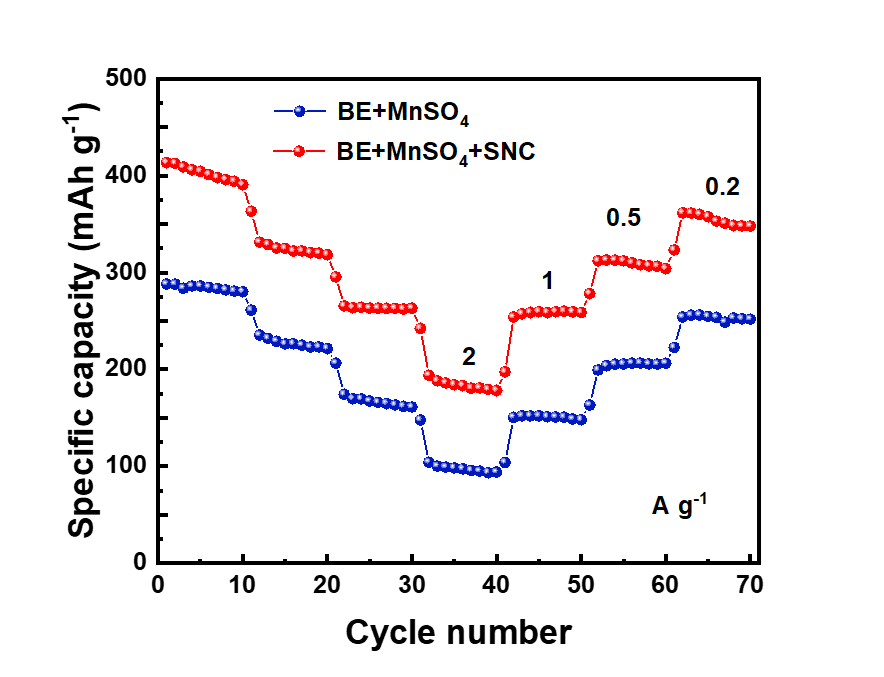


**Fig. S26** Rate performance of Zn-MnO_2_/CNT full cells in BE+MnSO_4_ and BE+MnSO_4_+SNC


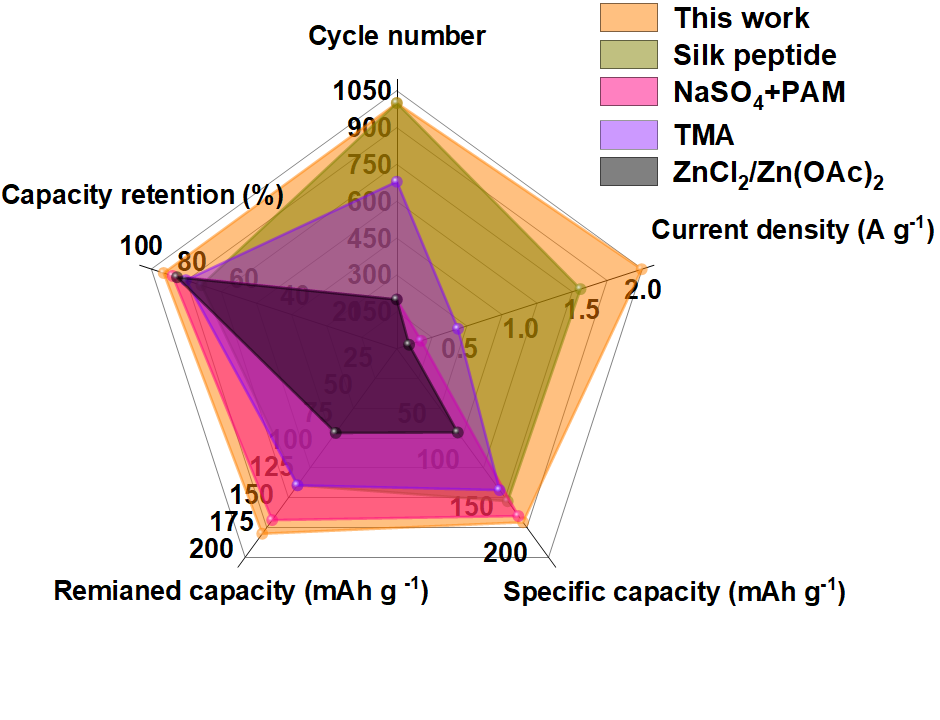


**Fig. S27** Multidimensional comparison of Zn-MnO_2_/CNT full cells assembled with BE+MnSO_4_+SNC with other previously reported literature based on electrolyte engineering


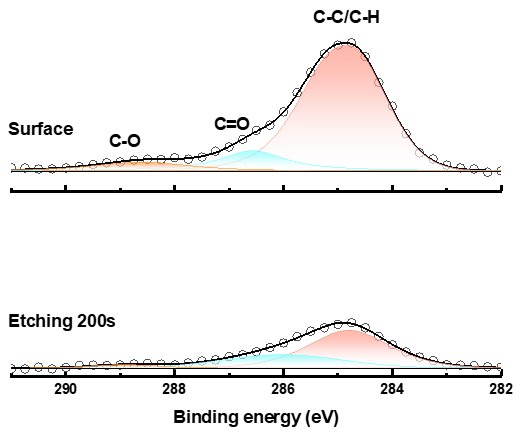


**Fig. S28** XPS spectra of C 1s on the surface of the Zn anode in the Zn|Zn symmetric cell after 30 cycles at 5 mA cm^-2^ and 5 mAh cm^-2^ with SNC
